# Mimicking the surface and prebiotic chemistry of early Earth using flow chemistry

**DOI:** 10.1038/s41467-018-04147-2

**Published:** 2018-05-08

**Authors:** Dougal J. Ritson, Claudio Battilocchio, Steven V. Ley, John D. Sutherland

**Affiliations:** 1 MRC Laboratory of Molecular Biology, Francis Crick Avenue, Cambridge Biomedical Campus, Cambridge, CB2 0QH UK; 2 Department of Chemistry, University of Cambridge, Lensfield Road, Cambridge, CB2 1EW UK; 3 Present Address: Syngenta Crop Protection, Process Research, Schaffhauserstrasse 101, CH-4332 Stein, Switzerland

## Abstract

When considering life’s aetiology, the first questions that must be addressed are “how?” and “where?” were ostensibly complex molecules, considered necessary for life’s beginning, constructed from simpler, more abundant feedstock molecules on primitive Earth. Previously, we have used multiple clues from the prebiotic synthetic requirements of (proto)biomolecules to pinpoint a set of closely related geochemical scenarios that are suggestive of flow and semi-batch chemistries. We now wish to report a multistep, uninterrupted synthesis of a key heterocycle (2-aminooxazole) en route to activated nucleotides starting from highly plausible, prebiotic feedstock molecules under conditions which mimic this scenario. Further consideration of the scenario has uncovered additional pertinent and novel aspects of prebiotic chemistry, which greatly enhance the efficiency and plausibility of the synthesis.

## Abstract (web summary)

There is still much debate on early Earth geochemical conditions affecting the chemistry of simple synthons that originated life. Here, the authors report an uninterrupted multistep synthetic route to 2-aminooxazole by means of flow chemistry equipment, mimicking a plausible early Earth (geo)chemical scenario.

## Introduction

The recalcitrant problem of the origin of life has many different facets, the most fundamental of all being the prebiotic synthesis of the minimum set of molecules which can lead to the first (living?) protocell. Intrinsically linked to this problem is the question of the physical place upon Earth where this synthesis took place, as the surrounding environment would have a direct bearing on the types of chemistry that were feasible^1^. This raises a question in itself—does one first consider a geochemical environment which one imagines could kick-start life and then (ideally) explore the chemistry which is possible within these bounds (geology first), or does one investigate a synthesis, which one hopes to be prebiotically plausible, and then try and fit that synthesis to an early Earth geological setting (chemistry first)?

An example of the geology first approach is deep-submarine vents, which have proven popular as a setting for the origin of life for many authors^2–7^. This popularity is due to several features of oceanic hydrothermal vents which appear to overlap with extant biology, and so a putative connection between abiotic and biotic worlds has been drawn. Over the last 30 years the model has undergone numerous refinements reaching a remarkable level of detail^8–16^. However, this would seem inconsistent with advances made in the laboratory, wherein only one productive synthetic experiment, conceivably consistent with vent conditions, has been reported—the conversion of CO and MeSH to (activated) acetate using Fe(Ni)S precipitates^17^, although these conditions might equally well have pertained to Earth’s surface. Neither nucleotides, amino acids nor lipids have been synthesised under vent conditions.

An example of the chemistry first approach to the origin of life is the formose reaction (Supplementary Fig. 1). Discovered in 1861 by Butlerow^18^, this reaction has appealed to prebiotic chemists in the modern era for several decades^19,20^ due to the fact that higher sugars, including ribose **1,** can be formed by heating aqueous formaldehyde **2** (a simple C_1_ feedstock molecule, CH_2_O) with calcium hydroxide, Ca(OH)_2_. Although there are severe, inherent problems with the formose reaction, which have caused many to question its prebiotic relevance (full critiques of the formose reaction can be found elsewhere^20,21^), some have attempted to control the non-selective and destructive nature of the reaction by the addition of various minerals, e.g., silicate and borate^22,23^. Initially this looks to be a logical step, but the additional demands now placed on the geochemical scenario must be met, and this has not proven possible to date (for a brief, in-depth discussion, see Supplementary Discussion). For example, Kim et al. attempted to use borate to direct the formose reaction to make and stabilise ribose en route to RNA. However, although borate stabilises ribose, it actually inhibits the formose reaction, and so eventually the authors resort to commencing from C_3_ sugars and formaldehyde **2**^23^. Although this did not afford ribose, it did produce *erythro-*branched and *thero-*branched pentoses, which it was proposed could form pentuloses via a molybdate-catalysed Bílik reaction. This was shown to take place at mildly acidic pH and (apparently) in the absence of borate. Whether or not the reaction would proceed in the presence of borate is likely a moot point, however, as the accumulation of significant concentrations of molybdenum, in its highest oxidation state, on early Earth has been deemed geologically implausible^24^. Thus, trying to shoehorn a geochemical scenario to fit a putative prebiotic synthesis would not appear to be a prudent strategy. Perhaps not surprisingly, taking an inflexible stance on chemistry or geology seems to place such demands on their respective counterparts, i.e., geological scenario or feasible chemistry, as to preclude progress.

**Fig. 1:**
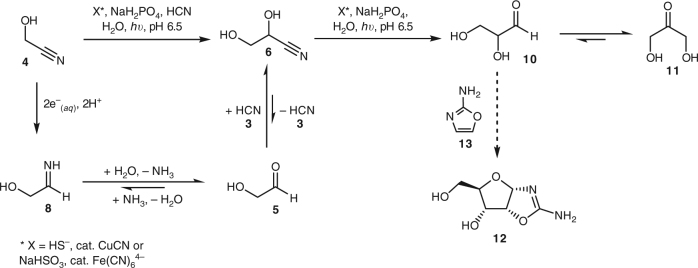
Photochemical reduction of cyanohydrins. Solvated electrons and/or H^•^ can efficiently reduce cyanohydrins. These reactions are promoted by Cu^+^ or Fe^2+^ ions and a sulfurous stoichiometric reductant under UV irradiation

Consequently, we have adopted a more flexible approach^25^, in which we first allowed the results of extensive chemical experimentation to loosely constrain geochemical conditions. We then explored other consequences of the inferred range of conditions on the synthetic chemistry and bootstrapped our way between chemistry and geology to optimise syntheses and further constrain the geochemical conditions. This led to the discovery of a chemical network that yielded activated pyrimidines, amino acids, phospholipid precursors and most of the components of the citric acid cycle^26,27^. Concomitantly, a picture of a geochemical environment unfolded which met the requirements of all reactions in the network, and seemed (to us at least) plausible. The geochemical scenario was dependent on schreibersite ((Fe,Ni)_3_P), HCN, hydrosulfide (HS^–^), copper and ultraviolet light under post-impact conditions. The action of UV light on catalytic amounts of copper(I) allows efficient reduction of cyanohydrins by hydrated electrons (e^–^_(*aq*)_) and/or H^•^ to form various aldehydes with HS^–^ being consumed as the stoichiometric reductant (Fig. 1)^26,27^. In a more recent refinement of this reaction, we have found that the photochemical reduction of nitriles can be performed equally effectively with catalytic ferrocyanide (Fe(CN_6_)^4−^) and bisulfite (HSO_3_^–^, Fig. 1)^28^. The presence of SO_2_ on early Earth would appear to have been probable^29,30^, if, according to a recent calculation, only present at a low, constant atmospheric level^31^. However, it has been pointed out that during periods of increased geothermal activity, spikes in atmospheric SO_2_ concentrations would have been expected^30,32^. In any event, due to the high solubility of SO_2_ in water and the low p*K*_a_ of sulfurous acid, HSO_3_^–^ could have been expected to be concentrated in ground water, even to the point of dryness^33^. Thus, given the presumed global abundance of Fe^2+^ ions, the Fe(CN_6_)^4−^/HSO_3_^−^ system may be a preferred means for achieving photoreductive homologation of hydrogen cyanide on early Earth. Interestingly, although atmospheric SO_2_ would be expected to absorb some UV light, it apparently permits the passage of light of the requisite wavelength to drive the photoreductive chemistry to the surface of Earth^31,32^.

**Fig. 2:**
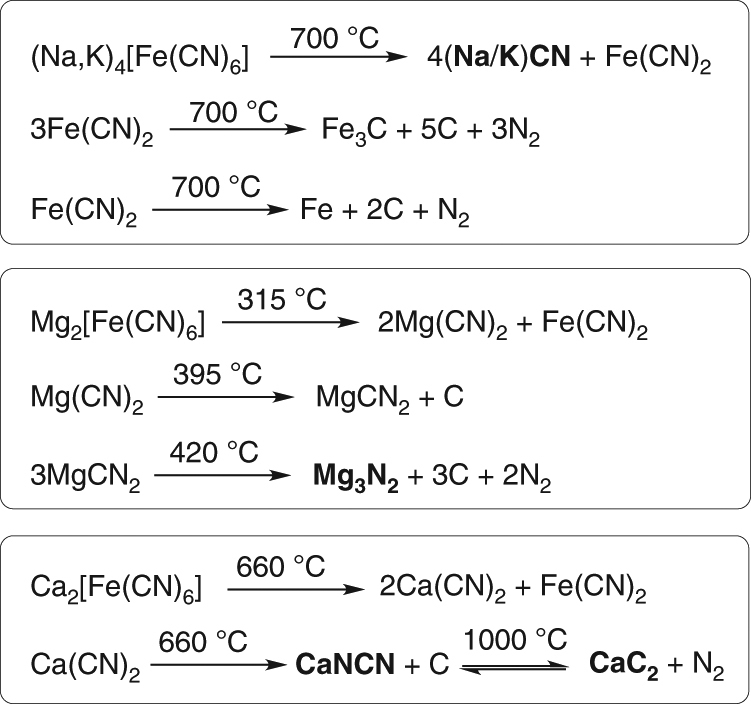
Thermal metamorphosis of ferrocyanide salts. The products formed by heating ferrocyanide can be altered simply by exchanging the counter-ion of ferrocyanide and/or varying the temperature to which the salt is heated

Key to our geochemical scenario^26,34^ is the Late-Heavy Bombardment: iron-rich meteorites provided FeS, (FeNi)S and (Fe,Ni)_3_P, the impact of falling bodies generated large quantities of atmospheric HCN **3** (the notation **3** is used to represent HCN and/or cyanide ion, NC^−^) through shock waves and electric discharge plasma^35^ and rain-in of **3** would have resulted in the corrosion of meteoritic metal inclusions providing cyanometallates, phosphate and HS^−^/H_2_S. In addition, the most abundant cyanometallate, ferrocyanide, when heated in the dry state, either from geothermal activity or further impact events, yields different products depending only on the nature of its counter ion (Fig. 2). Thus, the products from sodium/potassium, calcium and magnesium ferrocyanide are Na/KCN, calcium cyanamide + calcium carbide (CaNCN + CaC_2_) and magnesium nitride (Mg_3_N_2_), respectively—the exact feedstock molecules required to populate the protometabolic network previously reported^26^. Considering a landscape, distinct areas could have been rich in NaCN deposits or CaNCN deposits, based purely on the differences of abundance of Na^+^ and Ca^2+^ ions in those regions at the time of evaporation of water before thermal metamorphosis. At a later juncture, after rainfall, streams or rivulets could start to flow, picking up substrates, reagents and catalysts in a sequence determined by the order in which the dry-state repositories were encountered. Photochemistry and dehydration/hydration steps could ensue, and upon confluence of the streams mixing and further reaction could take place. This geochemical situation would offer the type of mechanism required for sequential delivery of reagents to allow constructive and selective synthesis of all the (proto)biomolecules previously reported^26^. There are numerous variations to this scheme, for example, fluvial activity could bring the reagents from different areas to merge in a pool. The pool could undergo drying and rehydration and occasionally, in heavy rain, overflow, mixing the contents with other pools. Although all the required chemical steps had been demonstrated by us in the laboratory, some were still sceptical that the prebiotic relevance of the scheme was not warranted as “the network cannot yet function in one pot without external interference”^36^. Notwithstanding the fact that the scheme is not meant to function in one pot, we wondered how we could best simulate the fluvial locale we envisaged without a hands-on approach. We were also keen to scrutinise the scenario again with a view to learning more ourselves. Below, we describe an uninterrupted synthesis of 2-aminooxazole from prebiotically plausible starting materials in an attempt to validate the plausibility of our geochemical scenario and the permissible prebiotic chemistry associated with that scenario. We found that bisulfite is an excellent all-round player, providing reducing power for the reduction of nitriles to aldehydes and the capacity to protect and concentrate the aldehydes, and therefore prebiotic amino acid and ribonucleotide precursors, yet, vitally, does not interfere with their syntheses.

## Results

### Evaluation of the photoreductive system

**Fig. 3:**
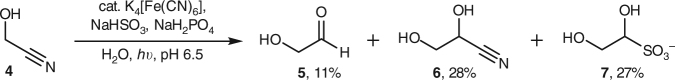
Photochemical reduction of **4** using the Fe^2+^/NaHSO_3_ system. Typical conditions: glycolonitrile **4** (30 mM), Na_2_SO_3_ (50 mM), NaH_2_PO_4_ (100 mM) and K_4_[Fe(CN)_6_] (5 mM) in H_2_O:D_2_O (9:1), pH adjusted to 6.5 with degassed HCl/NaOH and the solution was irradiated. Glyceronitrile **6** was presumed to be formed due to the liberation of cyanide in the work-up procedure, whereby paramagnetic Fe_x_(CN)_y_L_z_ species were removed by the addition of NaSH to allow NMR spectra to be acquired

It soon became obvious that flow chemistry—a phrase we construe less stringently than others do^37^—would be the ideal technique to simulate small streams flowing across Hadean/Archean Earth. Initial considerations of the optimal reductive system to be used (CuCN/HS^– ^+ *h*ν vs. Fe(CN)_6_^4–^/HSO_3_^– ^+ *h*ν) were determined by compatibility with the flow system. CuCN is insoluble in water, as is the black precipitate that forms upon contact with HS^–^ (presumably copper sulfides), and during the reduction of nitriles using this system, HS^–^ is ultimately oxidised to S_8_, which precipitates from aqueous solution. While this is of no consequence when considering a stream on ancient Earth, solids are not well-tolerated in flow chemistry due to the fine internal diameter of the tubing which is employed. Hence, we opted for the ferrocyanide-bisulfite system, as all starting materials, products and by-products are water soluble. As previously, we decided to begin the sequence from glycolonitrile **4** as the carbon source^27^. The presumed abundance of cyanide **3**, whether produced terrestrially via thermal metamorphosis of ferrocyanide to give K/NaCN **3** (Fig. 2) or atmospherically as a result of electric discharge^38^ and impact synthesis^35^, should mean that a large amount of formaldehyde **2** (also produced atmospherically^39^) would encounter **3** resulting in glycolonitrile **4**. Although the formation of **4** would be seen as detrimental to those wishing to exploit formaldehyde (vide supra) or HCN polymerisation chemistry, it affords the advantage of possessing a much higher boiling point than either of its components, i.e., glycolonitrile b.p. = 103 °C (16 mmHg)^40^ *cf*. HCN b.p. = 26 °C and CH_2_O b.p. = −19 °C, which would allow its concentration (to some degree) in ground water. Thus, a concentration of 30 mM of **4** did not seem to be an unreasonable starting point for investigation. We soon found promising conditions that gave excellent yields of glycolaldehyde **5** and its cyanide **6** and bisulfite **7** derivatives (>60%) after 2 h of irradiation under batch conditions (Fig. 3 and Supplementary Fig. 2), which suggested that good yields of **5** would be accessible in the flow chemistry experiments.

As discussed (vide supra), the accumulation of HSO_3_^–^ on the surface of Earth could have been extensive and even have resulted in dry bisulfite/sulfite-rich beds. Ferrocyanide and phosphate would have resulted from the corrosion of meteoritic schreibersite^26,34,41,42^, and could also be concentrated to dryness. Thus, in the first instance, we imagined a stream of glycolonitrile **4** meeting a stream of NaHSO_3_, K_4_[Fe(CN)_6_] and NaH_2_PO_4_, and the merged stream being exposed to sunlight. Of course, there are many variations of a theme one can think of that would result in a similar chemical system. For instance, a stream of **4** could run over a dry bed of NaHSO_3_, then a dry bed of NaH_2_PO_4_/K_4_[Fe(CN)_6_], dissolving up the salts in the process. This sequence would give a stream containing the same reactants although a far higher concentration of reagents could, in principle, be achieved. Alternatively, there could be a convergence of three streams, one of each containing glycolonitrile **4**, NaHSO_3_ and NaH_2_PO_4_/K_4_[Fe(CN)_6_], respectively. While contemplating these possibilities, a most pertinent point presented itself; if various streams were converging delivering reagents and substrates, after each interface the concentration of all solutes would be decreased. By how much would depend on the volume and flow rate of each stream, and, accordingly, may or may not still allow productive chemistry to occur. The number of permutations for the possible reaction sequences and reaction parameters was becoming excessive to investigate, but it was apparent that a means of concentration would lend credence to our geochemical scenario.

### Prebiotic protection and concentration of aldehydes

In view of this, our focus shifted to the glycolaldehyde-bisulfite adduct **7,** which was always formed, to some extent, in our reactions. If the reduced glycolonitrile **4** could be trapped as **7**, ideally in one pot, there was a chance that we could concentrate the sulfonate product. Indeed, the ability of bisulfite to trap aldehydes in a prebiotic setting has been demonstrated before by Pitsch et al., who used hydrotalcite sulfite to sequester aldehydes in the interstitial layers of the mineral^43^. By increasing the starting concentration of bisulfite, the reduction of glycolonitrile **4** to glycolaldehyde **5** with subsequent in situ addition of bisulfite became remarkably efficient. After 1.5 h irradiation of a solution of **4** (30 mM) in the presence of Na_2_SO_3_ (150 mM), NaH_2_PO_4_ (100 mM) and K_4_[Fe(CN)_6_] (17 mol%), **7** could be formed in ~80% yield (Supplementary Fig. 3), with ca. 5% of **4** remaining. Even at pH 6.5, with phosphate acting as a general acid-base catalyst, the addition of HSO_3_^–^ was competitive with the hydrolysis of glycolaldehyde imine **8**—the first product from the reduction of glycolonitrile **4**.

**Fig. 4:**
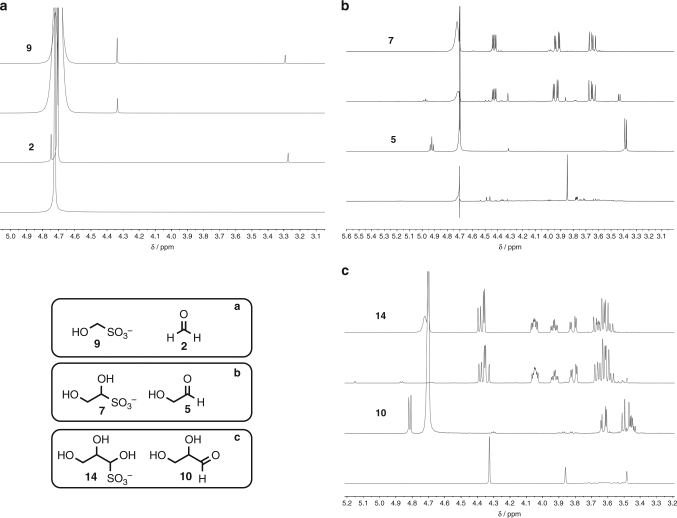
Protection and concentration of aldehydes. ^1^H NMR Spectra showing how aldehydes can be protected and concentrated under prebiotically plausible conditions via the intermediacy of their bisulfite adducts. **a** aldehyde = formaldehyde **2**; **b** aldehyde = glycolaldehyde **5**; **c** aldehyde = glyceraldehyde **10**. Upper spectra—solution of aldehyde, NaH_2_PO_4_ and HSO_3_^–^; centre upper spectra—as upper spectra after heating to dryness for 40 h then redissolving in D_2_O; centre lower spectra—as upper spectra without the addition of HSO_3_^–^; lower spectra—as centre lower spectra after heating to dryness for 40 h then redissolving in D_2_O (in **a**, the singlet observed at 3.3 ppm, upper and centre lower spectra, is MeOH, included in commercial formaldehyde as a stabiliser)

We then sought to exploit the kinetic and thermodynamic preference for bisulfite adduct formation to concentrate formaldehyde **2** and glycolaldehyde **5**—the two most abundant aldehydes, according to our scheme, and pertinent to prebiotic amino acid and nucleotide synthesis^26^. Hence, we took aqueous solutions of **2** and **5** in phosphate buffer (1 eq.) in the presence and absence of HSO_3_^–^ (1.2 eq.) at pH 6.5 and heated them open to the air at 55 °C for 40 h. The solid residues were redissolved in D_2_O and examined by ^1^H NMR spectroscopy (Fig. 4a, b). We found >55% of formaldehyde **2** was recovered as its bisulfite adduct **9**, hydroxymethanesulfonate (HMSA), after the evaporation process but, not unexpectedly, in the absence of bisulfite **2** evaporated completely (Fig. 4a). In the case of glycolaldehyde **5**, >90% of the aldehyde was recovered as its bisulfite adduct **7** or as its hydrate—presumably a small amount of bisulfite was lost in the drying process in the form of SO_2_ (Fig. 4b). In the absence of bisulfite, no glycolaldehyde **5** remained. A small amount of oligomerisation was observed (signals observed at 3.4–3.8 ppm and 4.3–4.5 ppm, Fig. 4b, lower spectrum) and some material was lost through evaporation, but the majority of the material reacted with atmospheric O_2_ to give glycolate (3.85 ppm, singlet, Fig. 4b, lower spectrum) and formate. On Hadean/Archean Earth, in the absence of oxygen, this latter pathway would not be possible, and the majority of **5** would be expected to be lost through oligomerisation and evaporation. Thus, forming the bisulfite adduct of an aldehyde not only allows it to be concentrated but also protects the aldehyde from aldol chemistry. When evaluated in these terms, we were keen to examine the ability of bisulfite to protect glyceraldehyde **10**, as its propensity to isomerise to the thermodynamically favourable ketone, dihydroxyacetone **11** (DHA), is well known^26^ and must be avoided if *ribo*-aminooxazoline **12** is to be accessed via direct reaction with 2-aminooxazole **13** (Fig. 1). When a solution containing glyceraldehyde **10** and bisulfite was submitted to the same dehydrating conditions, we were gratified to find that almost all **10** was recovered as its bisulfite adduct **14** and only ~4% was converted to DHA **11**. Yet in the absence of HSO_3_^–^, no **10** remained, again through reaction with atmospheric O_2_ and oligomerisation but mainly due to isomerisation to **11** (Fig. 4c, lower spectrum: singlet observed at 4.33 ppm due to DHA **11**, singlet observed at 3.85 ppm due to glycolate and singlet observed at 3.49 due to the hydrate of **11**. An enhanced spectrum can be found in Supplementary Fig. 4). We then investigated bisulfite’s protective capacity a little further. Glyceraldehyde **10** (100 mM), Na_2_SO_3_ (140 mM) and NaH_2_PO_4_ (100 mM) were dissolved in H_2_O and the solution was adjusted to pH 6.5. The tube was sealed and heated at 55 °C for 14 days, after which the solution was examined by ^1^H NMR spectroscopy. We observed that ~72% of the glyceraldehyde-bisulfite adduct **14** remained intact with ca. 9% DHA **11** and 19% methylglyoxal-bisulfite adduct **15** also present (Supplementary Fig. 5). Methylglyoxal results from the elimination of water from the enediol of the trioses **10** and **11**. If glyceraldehyde **10** was heated under the same conditions without the inclusion of HSO_3_^–^ for 18 h, **11** was formed in high yield with no **10** remaining (Supplementary Fig. 5). Interestingly, when DHA-bisulfite adduct **16** was heated under the same conditions ~50% of **15** had formed after only 24 h, and after 14 days virtually no **16** remained but a large amount of **15** was present (Supplementary Fig. 6).

**Fig. 5:**
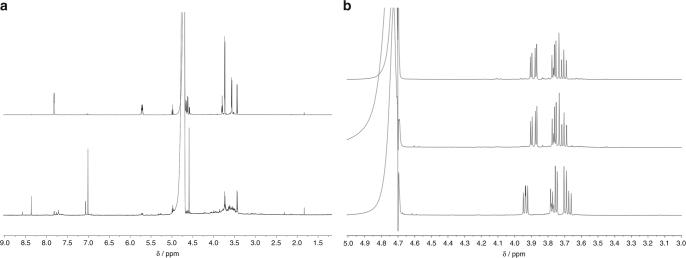
Protection of **5** at high pH and modified Strecker synthesis. ^1^H NMR Spectra showing the protection of glycolaldehyde **5** by bisulfite in the presence of NH_3_ and the Knoevenagel–Bucherer modified Strecker synthesis of serine aminonitrile. **a** Upper spectrum—**5** in H_2_O/D_2_O with NH_3_ at pH 9.2 after 10 min; lower spectrum—as upper spectrum after 7 days at room temperature. **b** Upper spectrum—**5** and Na_2_SO_3_ in H_2_O/D_2_O with NH_3_ at pH 9.2 after 10 min; centre spectrum—as upper spectrum after 7 days; lower—as centre spectrum with KCN added after 2 h, pH 9.2

Finally, we examined the behaviour of glycolaldehyde **5** in the presence of ammonia with and without added bisulfite at high pH (9.2). In the absence of HSO_3_^–^, a trace of decomposition is evident even after 10 min, and after 7 days little starting material remains (Fig. 5a). Once again, however, the reaction containing bisulfite exhibited excellent stability under the reaction conditions and after a week only a trace of degraded material was observed (Fig. 5b). At this juncture, it is appropriate to note that the majority of aldehydes formed in our scheme^26^ progress to become amino acids via Strecker synthesis (addition of NH_4_CN to the carbonyl). At first glance, it might appear that the formation of the aminoalkylsulfonates, from the corresponding aldehyde or aldehyde-bisulfite adduct, would thwart the synthesis of amino acids, yet the Knoevenagel–Bucherer modification of the Strecker synthesis^44^, proceeding precisely by these intermediates, has been known for over a century. Thus, addition of KCN (solution then re-adjusted to pH 9.2) to the glycolaldehyde-ammonia-bisulfite adduct, 1-amino-2-hydroxyethylsulfonate **17**, gave smooth conversion to serine aminonitrile within 2 h (Fig. 5b).

**Fig. 6:**
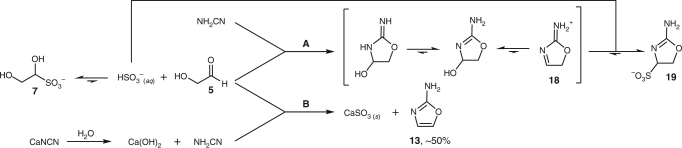
Reactions of **7** with NH_2_CN and CaNCN. The use of cyanamide, when attempting to form 2-AO **13** from **7** (pathway A), results in the undesirable reaction of displaced bisulfite with **18**, but using calcium cyanamide circumvents this problem by precipitating calcium sulfite (pathway B)

In keeping with the modified scenario, 2-aminooxazole **13** would now have to be made from the bisulfite adduct of glycolaldehyde **7**. When attempting the addition of cyanamide to **7**, two problems became apparent. First, the favourable kinetics and thermodynamics for the formation of the aldehyde’s bisulfite adduct coupled with cyanamide’s moderate nucleophilicity meant that the rate of addition of cyanamide to glycolaldehyde **5** was markedly hindered. Second, HSO_3_^–^ that was slowly displaced from glycolaldehyde-bisulfite adduct **7**, allowing cyanamide to react, then returned to displace hydroxide (presumably dissociatively via iminium **18**) and give aminooxazoline **19** quantitatively (Fig. 6, pathway A and Supplementary Fig. 7). Although there were several potential strategies which could be employed to circumvent this problem, the most succinct solution arose from considering the geochemical scenario we had proposed. Therein, cyanamide is derived from the thermal metamorphosis of Ca_2_[Fe(CN)_6_] (Fig. 2), the product being calcium cyanamide, CaNCN^45^. It transpires, most fortuitously, that calcium bisulfite is highly insoluble in H_2_O. Also, calcium cyanamide is immediately hydrolysed to Ca(OH)_2_ and NH_2_CN upon contact with water, so if a stream of glycolaldehyde-bisulfite adduct **7** ran over a deposit of CaNCN, or encountered another stream which had done so, the small amount of bisulfite present at equilibrium should precipitate as CaSO_3_ leaving free glycolaldehyde **5** and cyanamide in solution (Fig. 6, pathway B). Indeed, when CaNCN (3.5 eq.) was added to **7** (50 mM) and NaH_2_PO_4_ (50 mM) at pH 6.5 and heated to 45 °C for 12 h, 2-aminooxazole **13** was formed in ~50% yield (Supplementary Fig. 7).

### Uninterrupted flow synthesis of 2-aminooxazole 13

**Fig. 7:**
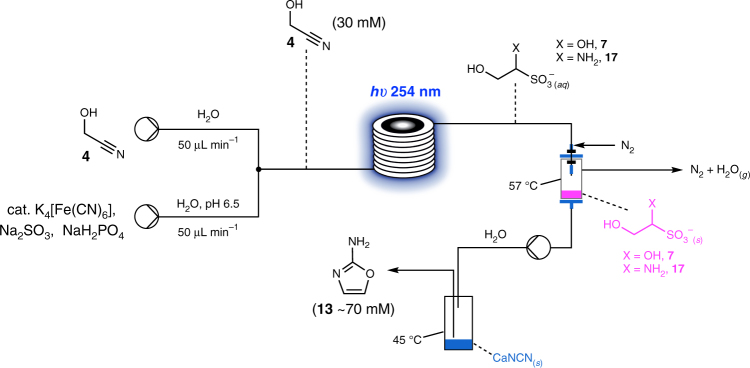
Flow chemistry set-up starting with glycolonitrile **4**. This arrangement was designed to simulate a possible geochemical environment on early Earth (see main text). Initial concentrations: glycolonitrile **4** (60 mM), Na_2_SO_3_ (200 mM), NaH_2_PO_4_ (50 mM) and K_4_[Fe(CN)_6_] (10 mM)

With the individual steps to 2-aminooxazole **13** now demonstrated, we set about stringing together an uninterrupted sequence to mimic a plausible environment on early Earth using flow chemistry equipment. Thus, a solution of glycolonitrile **4** was merged with a stream containing NaHSO_3_, K_4_[Fe(CN)_6_] and NaH_2_PO_4_ and pumped through a photochemical flow reactor, fitted with an Hg lamp, and an optimisation regime began (Fig. 7). Once conditions for the first step were resolved, we connected the outlet of the photochemical reactor to a solvent-switch system^46^ heated to 57 °C and with sufficient N_2_ pressure to remove H_2_O to the point of dryness (Fig. 7). After 100 min, the input stream was stopped and a small amount of H_2_O (typically 0.5–1.0 mL) was pumped into the collection chamber. The resulting solution was then pumped into a small vessel containing CaNCN heated at 45 °C for 3–6 h, after which time, the reaction was allowed to cool and sediment for several hours. An aliquot of the resulting clear, yellow/brown solution was then diluted in D_2_O and examined by ^1^H NMR spectroscopy. We were pleased to find 2-aminooxazole **13** had been formed in a very clean reaction with only one other major by-product (Supplementary Fig. 9). Furthermore, the concentration of **13** was now of the order of ~70 mM *cf*. 30 mM glycolonitrile **4** before the photochemical reduction.

With the intrinsic stability and involatility of hydroxyalkylsulfonates demonstrably apparent, we wondered if we could lower the concentration of glycolonitrile **4** and still obtain significant concentrations of 2-aminooxazole **13**. In a one-off, unoptimised experiment, we reduced the initial concentration of **4** to 4 mM (and other reagents in accordance), giving a solution of 2 mM in **4** before UV irradiation. Following the procedure as before, we obtained a final solution with concentration of **13** in the order of ~35 mM. Such a powerful mechanism for obtaining reasonably concentrated solutions of prebiologically relevant compounds warranted further investigation. Therefore (under batch conditions) we lowered the concentration of glycolonitrile **4** to 500 μM (approaching the limit of satisfactory ^1^H NMR spectroscopy) and irradiated the sample at 254 nm in the presence of NaHSO_3_ (2.3 mM), NaH_2_PO_4_ (1 mM) and K_4_[Fe(CN)_6_] (100 μM). At such low concentrations we anticipated a severe retardation in the rate of reduction, but counterintuitively, the reaction was almost complete after 20 min (Supplementary Fig. 10). Evidently, the reductant, H^•^ and/or e^–^_(*aq*)_, was diffusing very rapidly through H_2_O, possibly via a Grotthuss-like mechanism in the case of the former or hopping solvent cages in the case of the latter. This suggested, at higher concentrations, the rate of reduction was not the limiting factor but the supply of the reductant. If HSO_3_^–^ was acting as a filter for UV light, at higher concentrations Fe(CN)_6_^4–^ would be competing with sulfur for photons/unit volume, but at low concentration, photons were now, presumably, in excess. In view of these findings, we then examined the same reaction in the absence of ferrocyanide; as HSO_3_^–^ alone can function as a reasonable reducing agent under UV irradiation^28^. Although somewhat slower, the reduction of glycolonitrile **4** was >50% complete after only 1 h, and the only observable product was the bisulfite adduct **7** (Supplementary Fig. 11). Possibly the reaction could be run at lower concentrations still, which raised the possibility of atmospheric chemistry taking place in water droplets with **7** then raining onto Earth. In theory, a saturated solution of 2-aminooxazole **13** would be possible starting from very low concentrations of glycolonitrile **4**, but the fact that carbon is produced in the synthesis of CaNCN coupled with the fact that CaSO_3_ is insoluble means that this is not the case. The suspension that forms upon reaction must sediment, and above a certain concentration this appears unlikely to occur under our laboratory conditions. From the current studies, we would put an upper limit of ~150 mM for solutions of 2-aminooxazole **13** formed via the described chemistry.

**Fig. 8:**
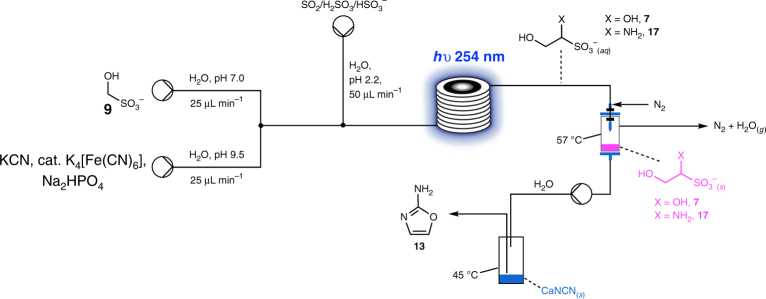
Flow chemistry set-up starting with HMSA **9**. This arrangement was designed to mimic the second variant of our geochemical scenario (see main text)

If SO_2_ was abundant, it follows that HMSA **9** would have accumulated on Earth, and consequently, we wondered if there was a way we could tap into what should have been a plentiful source of a C_1_-feedstock molecule. At neutral pH in the presence of an equimolar concentration of HCN **3**, equilibrium lies in favour of glycolonitrile **4** over **9**. However, exchange is extremely sluggish, and even after 1 week equilibrium is not reached (Supplementary Fig. 12). Once again, we considered the reagents available to us defined by our geochemical model and considered a stream containing HMSA **9** moving over an area where K/NaCN had been deposited (Fig. 2). At high pH, the exchange of NC^–^ and HSO_3_^–^ is much faster, and equilibrium is attained within 15 min at pH 9.2 (3:1, glycolonitrile:HMSA, Supplementary Fig. 13). If SO_2_/SO_2_.xH_2_O/HSO_3_^–^ was rained into the alkaline stream of glycolonitrile **4,** the pH could be dropped instantly. We attempted to recreate this situation with the flow chemistry apparatus by merging three streams: one containing HMSA **9** at pH 7.0, one containing KCN, Na_2_HPO_4_ and K[Fe(CN)_6_] at pH 9.5 and one containing NaHSO_3_ at pH 2.2 (Fig. 8, although the p*K*_a_ of sulfurous acid is ~1.8 there would have been some buffering by CO_2_/HCO_3(*aq*)_, so we proposed a pH of 2.2 not to be unrealistic). The reaction was pumped through the UV reactor, concentrated and reacted with CaNCN as before to furnish a solution that was >60 mM in 2-aminooxazole **13** *cf*. a theoretical maximum 30 mM in glycolonitrile **4** after confluence of the three streams. Again, there are multiple variations of a theme which should result in the same chemical outcome. For example, atmospheric CO_2_ could buffer the solution of **4** formed after a stream of HMSA **9** had encountered K/NaCN, which may take a little more time but would certainly be expected. Or, a stream of **9** and HCN could have come into contact with alkaline streams/evaporite beds—resulting from the serpentinisation of ultramafic rock—which would temporarily raise the pH and facilitate the exchange of NC^–^ and HSO_3_^–^. Once again, the number of permutations of reaction sequences, coupled with all the variable reaction parameters, was prohibitive to investigate without high throughput, multiplet flow apparatus, but we were satisfied a convincing demonstration of the uninterrupted synthesis of 2-aminooxazole **13** starting from C_1_-building blocks was complete.

## Discussion

Pinning down a precise geochemical scenario which could lead down a predisposed trajectory towards life is understandably challenging. In the described work, we have sought to take the initial steps to a “continuous”^47^ synthesis of ribonucleotides from prebiotically available C_1_ or C_2_-starting materials. During the course of the investigation, we found that bisulfite, previously used to reduce HCN **3** to glycolonitrile **4**, glyceronitrile **6** and glyceraldehyde cyanohydrin^28^, could be used to form glycolaldehyde-bisulfite adduct **7** directly from **4** in high yield. We had been cognisant of the fact that if the Cu^+^/HS^–^ photochemistry previously described^26,27^ occurred at a time when cyanide concentrations were low, the resulting aldehydes would be left in solution and degradation would be expected to take place over a period of days/weeks. However, we have found that bisulfite adduct formation is an extremely effective way of stabilising aldehydes, almost halting degradation pathways completely. Even in the presence of ammonia at high pH, the aminosulfonates are incredibly robust yet, crucially, allow facile synthesis of aminonitriles in the presence of cyanide **3**. Furthermore, thanks to the low p*K*_a_ of the conjugate acid of the sulfonate that is formed, the bisulfite adducts can be concentrated to the point of dryness. In view of the proposed geochemical scenario, a concentration mechanism of volatile intermediates would appear to be an essential requirement, and has been realised by adopting recent suggestions concerning Earth’s primitive atmosphere^30–33^. Finally, glycolaldehyde **5** is required for the synthesis of 2-aminooxazole **13**, but in this case, bisulfite interferes with 2-aminooxazole **13** synthesis. The simplest solution to this problem—removal of bisulfite from equilibria by precipitation as CaSO_3(*s*)_—became apparent by considering the reagent (CaNCN) that our geochemical scenario suggested, rather than the reagent we normally take from the fridge (NH_2_CN).

**Fig. 9:**
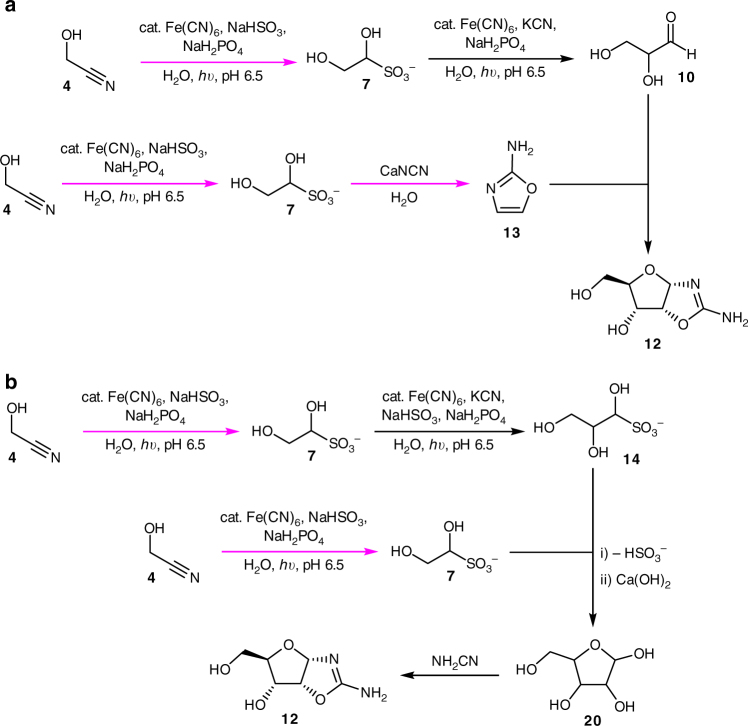
The two viable routes to *ribo-*aminooxazoline **12** starting from **4**. All steps have been demonstrated under batch conditions, but those steps depicted in magenta have been performed in flow conditions in a “continuous”^48^, uninterrupted manner starting from glycolonitrile **4** or HMSA **9**. **a** Route to **12** via the addition of 2-AO **13** to glyceraldehyde **10**. **b** Route to **12** via aldol reaction of **5** and **10** after their liberation from **7** and **14**, respectively

Clearly, there is a long way to go from the current position to making oligonucleotides (let alone a protocell) in an uninterrupted synthetic sequence, yet the results presented here show that the first steps along that road appear to be predisposed in favour of the necessary chemical reactions and physical processes. Furthermore, the solutions to the problems we encountered thus far have been intertwined with the geological and chemical scenario we have proposed, and allow a clean, telescoped synthesis of 2-aminooxazole **13**. Thus, the generalisation that “if you have a pool of chemicals and pump energy in, you don’t get life, you get asphalt” appears not to be correct if one is examining the right type of reaction sequence^48^. As alluded to, the number of variables at each stage is manifold, and as the scheme progresses, there appear to be alternate routes to arrive at the same intermediates. For example, the next phase should be to evaluate an uninterrupted synthesis of glyceraldehyde **10**, which can react with **13** en route to the seemingly critical, crystalline intermediate *ribo-*aminooxazoline **12**^49^ (Fig. 9a). However, it may be possible that a mixture of the bisulfite adducts of glycolaldehyde **5** and glyceraldehyde **10** could be formed, concentrated and then used in a Ca(OH)_2_-mediated aldol reaction to give pentose sugars that finally undergo reaction with cyanamide to arrive at the same, key intermediate **12** (Fig. 9b). Although there are multitudinous variations, they are variations on a theme and all fall under the umbrella of the geochemical scenario outlined here and elsewhere^26,34^. However, it is not the authors wish or intent to persuade the reader that all roads must have led to Rome. In fact, we caution the opposite. While there are many small, and large, variances of sequence that could have still permitted a route to life, there are far, far more that would not. For example, if in one location a stream containing HMSA **9** ran over a deposit of CaNCN before K/NaCN was encountered, the mixture would have been converted to an insoluble polymer (Powner and Sutherland, unpublished).

There is a general consensus that the individual reactions leading to life should be robust and high yielding, or predisposed. However, it is unclear whether a sequence of such reactions leading to life could occur under one set of conditions, or whether changes of conditions and merger of different reaction streams etc. is required. The latter would make life less probable than the former, and the results reported herein suggest the latter. It follows that the sequence of events that led to life must have been highly contingent and the origin of life as we know it could have been a low probability event.

## Methods

### General procedure for UV irradiation experiments in batch

In 10% D_2_O/H_2_O (degassed), NaH_2_PO_4_.2H_2_O and Na_2_SO_3_ were dissolved and the solution was adjusted to pH 6.5 with degassed HCl/NaOH. The volume was made up to the desired amount using degassed 10% D_2_O/H_2_O and glycolonitrile **4** and K_4_[Fe(CN)_6_] (if required) were added. The solution was transferred to a quartz cuvette, stoppered and irradiated for the desired length of time. A sample was then removed, to which NaSH.xH_2_O (typically 1–2 mg) was added, centrifuged and analysed by ^1^H NMR spectroscopy.

### General procedure for dry-down experiments

In an Eppendorf, the aldehyde (0.050 mmol) was dissolved in a solution of NaH_2_PO_4_.2H_2_O (8 mg, 0.050 mmol) and Na_2_SO_3_ (8 mg, 0.060 mmol, if required) in degassed H_2_O (0.6 mL) and the pH adjusted to 6.5 with degassed HCl/NaOH. The volume was made up to 1 mL with degassed H_2_O and heated to 55 °C, with stirring, open to the air for 40 h. Although the samples were dry in under 40 h, we wished to test the stability of the bisulfite adducts in the dry state also. The residue was dissolved in D_2_O and analysed by ^1^H NMR spectroscopy.

### Stability of **10**, **14** and **16** in solution

In an Eppendorf, the carbonyl compound (9 mg, 0.100 mmol) was dissolved in a solution of NaH_2_PO_4_.2H_2_O (16 mg, 0.100 mmol) and Na_2_SO_3_ (18 mg, 0.140 mmol, if required) in degassed 10% D_2_O/H_2_O (0.6 mL), and the pH adjusted to 6.5 with degassed HCl/NaOH. The volume was made up to 1 mL with degassed 10% D_2_O/H_2_O, sealed, then heated to 55 °C for the desired time. A sample was then removed and analysed by ^1^H NMR spectroscopy.

### Procedure for checking the stability of **5** and **7** at high pH

In an Eppendorf, glycolaldehyde **5** (3 mg, 0.050 mmol) was dissolved in a solution of Na_2_SO_3_ (7 mg, 0.060 mmol, if required) in degassed 10% D_2_O/H_2_O (0.5 mL) and c.NH_4_OH (100 μL) added. The pH was adjusted to 9.2 with degassed HCl/NaOH, the volume was made up to 1 mL with degassed 10% D_2_O/H_2_O and the reaction was sealed. ^1^H NMR spectroscopy was performed at desired time points.

### Knoevenagel–Bucherer–Strecker synthesis of serine nitrile

After 7 days, to the reaction above containing bisulfite (500 μL), KCN **3** (3 mg, 0.050 mmol) was added. The pH increased to 9.3 but was readjusted to 9.2. ^1^H NMR spectroscopy was performed at desired time points.

### Reaction of **7** with NH_2_CN and CaNCN

Glycolaldehyde **5** (3 mg, 0.050 mmol) was dissolved in a solution of Na_2_SO_3_ (8 mg, 0.060 mmol) and NaH_2_PO_4_.2H_2_O (8 mg, 0.050 mmol) in degassed 10% D_2_O/H_2_O (0.6 mL), and the pH was adjusted to 6.5 with degassed HCl/NaOH. The volume was made up to 1 mL using degassed 10% D_2_O/H_2_O, and either cyanamide or calcium cyanamide were added (0.175 mmol). The reaction was heated to 45 °C for the desired time and a sample was removed and examined by ^1^H NMR spectroscopy (in the reaction using CaNCN, the Eppendorf was briefly centrifuged to obtain a clear solution for NMR spectroscopy. The resulting pellet was resuspended before continuing the reaction).

### Reaction of **13** with bisulfite

A solution of Na_2_SO_3_ (8 mg, 0.060 mmol) and NaH_2_PO_4_.2H_2_O (8 mg, 0.050 mmol) in degassed 10% D_2_O/H_2_O (1 mL) was adjusted to pH 6.5 and 2-aminooxazole **13** (4 mg, 0.050 mmol) was added. The reaction was sealed and heated overnight at 45 °C before being analysed by ^1^H NMR spectroscopy.

### Flow chemistry procedure starting from glycolonitrile **4**

In one vessel, glycolonitrile **4** (55%, 119 μL, 1.20 mmol) was dissolved in degassed H_2_O (20 mL) and kept under N_2_ atmosphere. A second vessel was charged with degassed H_2_O (15 mL), Na_2_SO_3_ (504 mg, 4.00 mmol) and NaH_2_PO_4_ (120 mg, 1.00 mmol), and the pH was adjusted to 6.5 with degassed HCl/NaOH. The volume was made up to 20 mL with degassed H_2_O and K_4_[Fe(CN)_6_].3H_2_O (84 mg, 0.200 mmol) was added and the solution was kept under N_2_ atmosphere. The two solutions were pumped at a rate of 50 μL min^−1^ each and merged via a T-piece. The solution was then pumped through a photochemical flow reactor at 25 °C (overall flow rate 100 μL min^−1^, 10 mL reactor coil (100 min total irradiation time), and a backpressure regulator was fitted at the output and adjusted so a pressure of ~2 bar was maintained) [the output was checked at the desired time points via the addition of NaSH.XH_2_O (1–2 mg), doping with D_2_O and acquiring solvent suppression ^1^H NMR spectra]. The reactor output was then delivered to an in-line solvent-switch system^46^ heated at 57 °C with an influx of N_2_ set at a pressure such that the output was concentrated to dryness. After 100 min of collection, the input was ceased, and degassed H_2_O (typically 0.5–1 mL) was either pumped or syringed into the solvent switch. The chamber was agitated gently to facilitate dissolution of the solids, and the solution was then pumped into an Eppendorf containing CaNCN (90%, 71 mg, 0.797 mmol) and stirrer bar. The Eppendorf was sealed and the suspension was stirred and heated at 45 °C for the desired time, after which the reaction was allowed to cool to room temperature and sediment for several hours. An aliquot of the clear solution was then dissolved in D_2_O and examined by ^1^H NMR spectroscopy.

### Flow chemistry procedure starting from HMSA **9**

In one vessel, Na_2_SO_3_ (302 mg, 2.40 mmol) and formaldehyde (37%, 180 μL, 2.40 mmol) were dissolved in degassed H_2_O (15 mL), and the pH adjusted to 7.0 with degassed HCl/NaOH. The volume was made up to 20 mL with degassed H_2_O and kept under N_2_ atmosphere (solution A). In a second vessel, KCN (182 mg, 2.80 mmol), NaH_2_PO_4_ (240 mg, 2.00 mmol) and K_4_[Fe(CN)_6_].3H_2_O (167 mg, 0.400 mmol) were dissolved in degassed H_2_O (15 mL) and the pH adjusted to 9.5 with degassed HCl/NaOH. The volume was made up to 20 mL with degassed H_2_O and kept under N_2_ atmosphere (solution B). A third vessel was charged with Na_2_SO_3_ (328 mg, 2.60 mmol) and degassed H_2_O (15 mL), and the pH adjusted to 2.2 with degassed HCl/NaOH. The volume was made up to 20 mL with degassed H_2_O and kept under N_2_ atmosphere (solution C). Solutions A and B were pumped at a rate of 25 μL min^−1^ each and merged via a T-piece. The resulting reaction stream was then merged via another T-piece with solution C, pumped at 50 μL min^−1^, and passed through the photochemical reactor at 25 °C (overall flow rate 100 μL min^−1^, 10 mL reactor coil (100 min total irradiation time), and a backpressure regulator was fitted at the output and adjusted so a pressure of ~2 bar was maintained) [the output was checked at the desired time points via the addition of NaSH.XH_2_O (1–2 mg), doping with D_2_O and acquiring solvent suppression ^1^H NMR spectra]. The solution was then pumped into an in-line solvent-switch system^46^ heated at 57 °C with an influx of N_2_ set at a pressure such that the output was concentrated to dryness. After 100 min of collection, the input was ceased and degassed H_2_O (typically 0.5–1 mL) was either pumped or syringed into the solvent switch. The chamber was agitated gently to facilitate dissolution of the solids, and the solution was then pumped into an Eppendorf containing CaNCN (90%, 71 mg, 0.797 mmol) and a stirrer bar. The Eppendorf was sealed and the suspension was stirred and heated at 45 °C for the desired time, after which the reaction was allowed to cool to room temperature and sediment for several hours. An aliquot of the clear solution was then dissolved in D_2_O and examined by ^1^H NMR spectroscopy.

### Equilibration of KCN, HSO_3_^–^ and **2** at neutral pH

A solution of Na_2_SO_3_ (6.3 mg, 0.060 mmol), NaH_2_PO_4_.2H_2_O (8 mg, 0.050 mmol) and formaldehyde **2** (37%, 3.8 μL, 0.050 mmol) in degassed 10% D_2_O/H_2_O (0.4 mL) was adjusted to pH 6.5 with degassed HCl/NaOH. The volume was made up to 0.5 mL with degassed 10% D_2_O/H_2_O. KCN (3.3 mg, 0.050 mmol) was dissolved in degassed 10% D_2_O/H_2_O (0.4 mL) and adjusted to pH ~7 with degassed HCl/NaOH. The volume was made up to 0.5 mL with degassed 10% D_2_O/H_2_O. The two solutions were then mixed, sealed and monitored by ^1^H NMR spectroscopy.

### Equilibration of KCN, HSO_3_^–^ and **2** at high pH

A solution of Na_2_SO_3_ (6.3 mg, 0.060 mmol), NaH_2_PO_4_.2H_2_O (8 mg, 0.050 mmol) and formaldehyde **2** (37%, 3.8 μL, 0.050 mmol) in degassed 10% D_2_O/H_2_O (0.4 mL) was adjusted to pH 9.2 with degassed HCl/NaOH. The volume was made up to 0.5 mL with degassed 10% D_2_O/H_2_O. KCN (3.3 mg, 0.050 mmol) was dissolved in degassed 10% D_2_O/H_2_O (0.4 mL) and adjusted to pH 9.2 with degassed HCl/NaOH. The volume was made up to 0.5 mL with degassed 10% D_2_O/H_2_O. The two solutions were then mixed, sealed and monitored by ^1^H NMR spectroscopy.

### Data availability

The authors declare that all data supporting the findings of this study are available within the paper and its Supplementary Information files or are available from the authors upon reasonable request.

## Electronic supplementary material

Supplementary Information
