# Supplementary material for: Mimicking the surface and prebiotic chemistry of early Earth using flow chemistry
Source: Nat Commun. 2018 May 8;9:1821. doi: 10.1038/s41467-018-04147-2 (PMC5940729; doi:10.1038/s41467-018-04147-2)
Supplement: Supplementary file 1 — Supplementary Information [file 41467_2018_4147_MOESM1_ESM.pdf]

Supplementary Information for

Mimicking the surface and prebiotic chemistry of early Earth using flow chemistry

Dougal J. Ritson[1], Claudio Battilocchio[2], Steven V. Ley[2] and John D. Sutherland[1]

[1]MRC Laboratory of Molecular Biology, Francis Crick Avenue, Cambridge Biomedical Campus, Cambridge CB2 0QH, UK. [2]Department of Chemistry, University of Cambridge, Lensfield Road, Cambridge, CB2 1EW, UK.

Supplementary Discussion

The formose reaction

As can be seen in Supplementary Fig. 1 the formose reaction can provide access to ribose **1**. Furthermore, the reaction can proceed round an autocatalytic cycle (highlighted in magenta) fixing CH_2O **2** at an exponential rate and generating more complex molecules. The cycle is autocatalytic in glycolaldehyde **5**, because for each turn of the cycle double the number of molecules of **5** are produced relative to the number of molecules of **5** which entered the cycle. However, the reaction is completely unselective because the basic conditions employed mean that any carbonyl that can enolise does so, and as hydroxymethylation (addition to formaldehyde **2**) is faster than reprotonation, random oligomerisation ensues. Furthermore, under the basic conditions retro-aldol reactions are facile, as are Cannizzaro reactions, which leads to the vast array of products that the formose reaction produces[1]. However, the provenance of ribose by such a cheap means has lured some to investigate a means of controlling the chaotic and destructive nature of the reaction conditions by addition of minerals[2,3]. As mentioned in the main text (see Introduction), the additional steps/reactants must then be accounted for in the overall geochemical scenario. To this end, a geochemical scenario was proposed recently wherein the alkaline water from serpentinisation erodes borate minerals from igneous rocks and runs into an intermontane basin which rapidly, that is before atmospheric CO_2 neutralises or acidifies the solution (the presumed relatively high atmospheric concentration of CO_2 on primitive Earth has been pointed out and estimated a number of times, but for a recent calculation see Todd *et al.*[4]), dries out, leaving behind alkaline borate deposits[5]. Formaldehyde containing rainwater rehydrates this mixture to provide a localised environment that is basic and contains **2** and borate (which it is proposed will control the formose reaction to yield ribose[3]). Notwithstanding the problem of concentration of **2** in rainwater (the cut-off point, of concentration of formaldehyde, for reasonable formose reaction is in the range of 10-100 mM[6,7], although more concentrated solutions are typically employed, > 100 mM[8,9]), Kim *et al.* point out that the

presence of borate actually inhibits the formose reaction and only Cannizzaro reaction of **2** takes place[3]. To overcome this impasse, modified formose reactions have been explored. For example, glycolaldehyde **5**, also reported in spark discharge experiments[10], was used in excess relative to formaldehyde **2** and although ribose **1** was reported to be observed in ^{13}C NMR spectra it was not present in sufficient quantities to be isolated. In any event, as the authors noted, generating an excess of **5** over **2** atmospherically is not likely, and so a much more reasonable geochemical scenario was to be investigated - "we examined how borate might guide and possibly inhibit cycles that fix HCHO on a large scale, where prebiotic glycolaldehyde would be a catalyst"[3]. It is somewhat puzzling then, that none of the subsequent reactions involve formaldehyde **2** and glycolaldehyde **5**. Instead, reactions involving glyceraldehyde **10**, or DHA **11**, with **2** in the presence of borate were examined and were found to give C2-branched pentoses in ~10% yield. Setting aside the fact that the authors do not demonstrate how C_3 sugars can be made in made in high yield (or in a clean, low yielding reaction and then concentrated) under prebiotically plausible conditions consistent with their geochemical scenario (the approach of taking a purified, advanced intermediate and using it in any desired concentration has been criticised caustically by some[11] and described as a "discontinuous synthesis model" by others[12]), it was suggested that a retro-aldol reaction of the branched pentoses using $Ca(OH)_2$ could give **10** and/or **11** and **5** which would then fix more formaldehyde **2**. As $Ca(OH)_2$ was used for the initial aldol reaction, this is in keeping with the geochemical scenario, but the borate which is required to make and stabilise the C2-branched pentoses inhibits the Ca-catalysed retro-aldol reaction[3]. Kim *et al.* seek to mitigate this problem by using molybdate (MoO_4^{2-}) to catalyse a Bìlik reaction at moderately acidic pH (which could occur from atmospheric CO_2 buffering) to give pentuloses[3]. Geochemically, this would seem a tenuous proposal for several reasons: 1) molybdenum is required in its highest oxidation state but Earth's crust/magma were reducing, 2) according to recent estimates the only molybdate mineral likely to have been accessible on early Earth was the highly stable mineral molybdenite (MoS_2)[13] and 3) it is thought hundreds of

millions, if not billions, of years would have been required to concentrate molybdenum to a level at which it could differentiate itself from other minerals[13]. There would appear to be chemical incongruities also, as the Bìlik reaction involving Mo^{6+} and the two C2-branched pentoses (prepared synthetically) was apparently carried out in the absence of borate (the authors state that the products of this Bìlik reaction, xylulose and ribulose, will isomerise under the conditions to xylose and ribose, respectively. Although the transformations are not demonstrated, reference is made to the literature. However, the cited articles demonstrate the ability of borate to push ketose-aldose equilibria in favour of ketose sugars[14,15,16,17,18]). Whether borate slows or prevents molybdate functioning as intended is not clear. Furthermore, a demonstration of the aldol reaction of a C_3 sugar with formaldehyde **2** in the presence of borate *and* molybdate giving the C2-branched pentoses is not reported, nor that molybdate can still function as a catalyst for the Bìlik reaction after the high pH required for the aldol reaction. Thus, the minerals which are used to overcome individual, isolated chemical problems initially, seem to be irreconcilable when attempting to fit them to a realistic early Earth geochemical scenario. Whether an uninterrupted, selective synthesis of ribose **1**, starting from formaldehyde **2** or from **2** and catalytic glycolaldehyde **5**, will be achieved remains to be seen, but the number of problems which must be solved to make ribose in a synthetic scheme of this ilk appears to be growing rather than lessening.

Supplementary Methods

General Experimental

Reagents and solvents were bought from Sigma-Aldrich, Alfa Aesar and Santa Cruz Biotechnology and were used without further purification. Batch photochemical reactions were carried out using a Rayonet RPR-200 photochemical reactor chamber with cooling fans switched on and fitted with low pressure Hg lamps (principle emission 254 nm) in Spectrosil quartz cuvettes. Photochemical flow reactions were performed using a Vapourtec E-series platform equipped with the UV-150 module. This module consists of a temperature controlled irradiation chamber where a transparent fluorinated ethylene polymer (FEP) reactor (1 mm i.d., 10 mL, S4 PN: 50-1287) is coiled around a low pressure Hg lamp (principle emission at 254 nm). A Mettler Toledo SevenEasy pH Meter S20 was used to monitor pH and deoxygenation of the batch reactions was achieved by sparging Argon through the solution for 20 min before use. Deoxygenation of large volumes of water, used in flow chemistry experiments, was achieved by 3 x high vacuum/N_2 cycling, then sparging with N_2 for ~35 min. ^{1}H NMR Spectra were acquired using a Bruker Ultrashield 400 Plus, Bruker Ascend 400 or Bruker-AC 400 (at 400.1 MHz) with the residual solvent peak as the internal reference (HOD = 4.70). Samples consisting of H_2O/D_2O mixtures were analysed using HOD suppression to collect ^{1}H NMR data.

Supplementary Figures

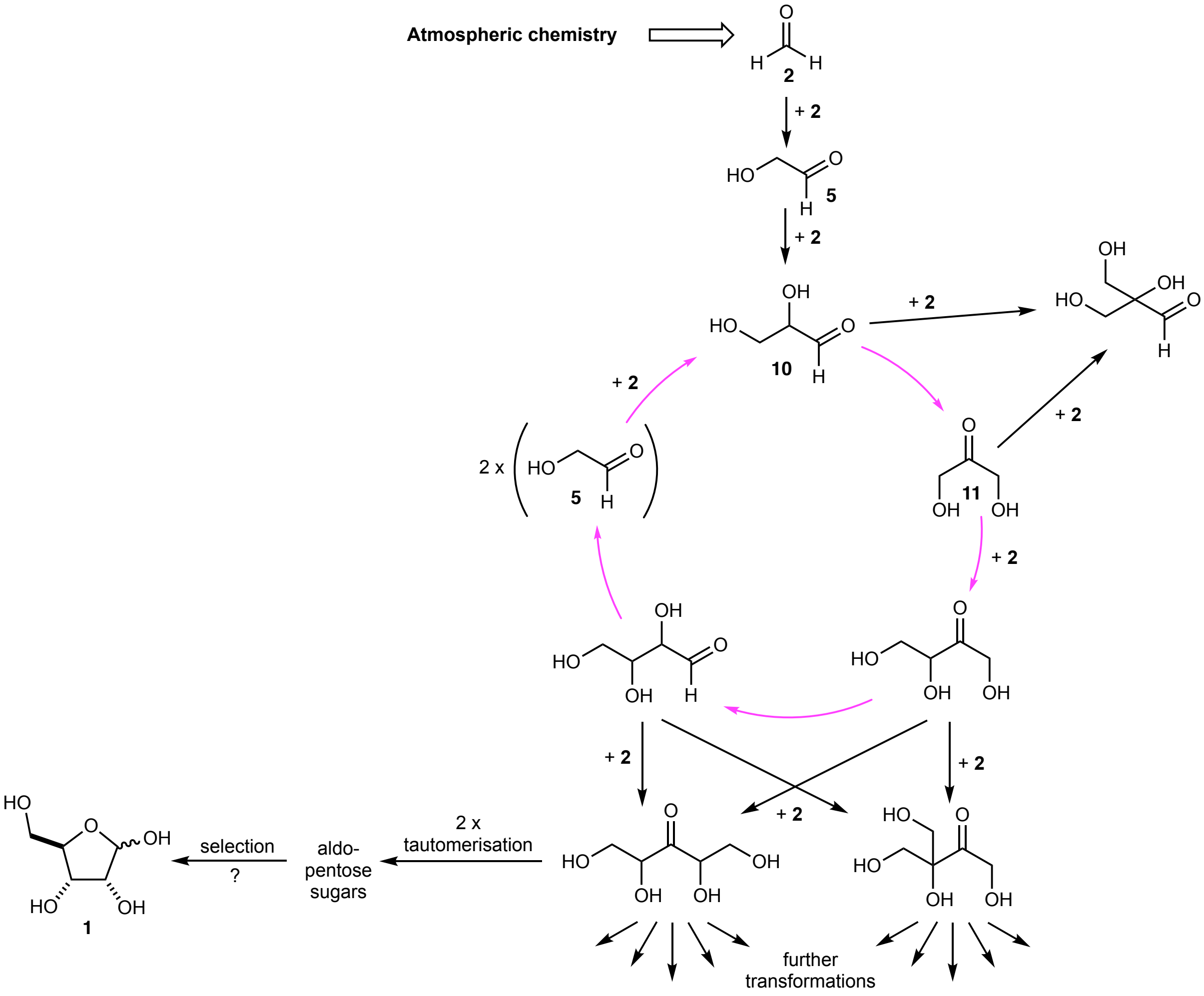

Supplementary Fig. 1 Initial stages of the formose reaction. Formaldehyde **2** is polymerised to make a large number of products indiscriminately (the manifold pathways and products are not included for clarity). Although ribose **1** is one of the many products of the formose reaction, how it could be selected from all the other sugars (or even the aldopentoses) is unknown.

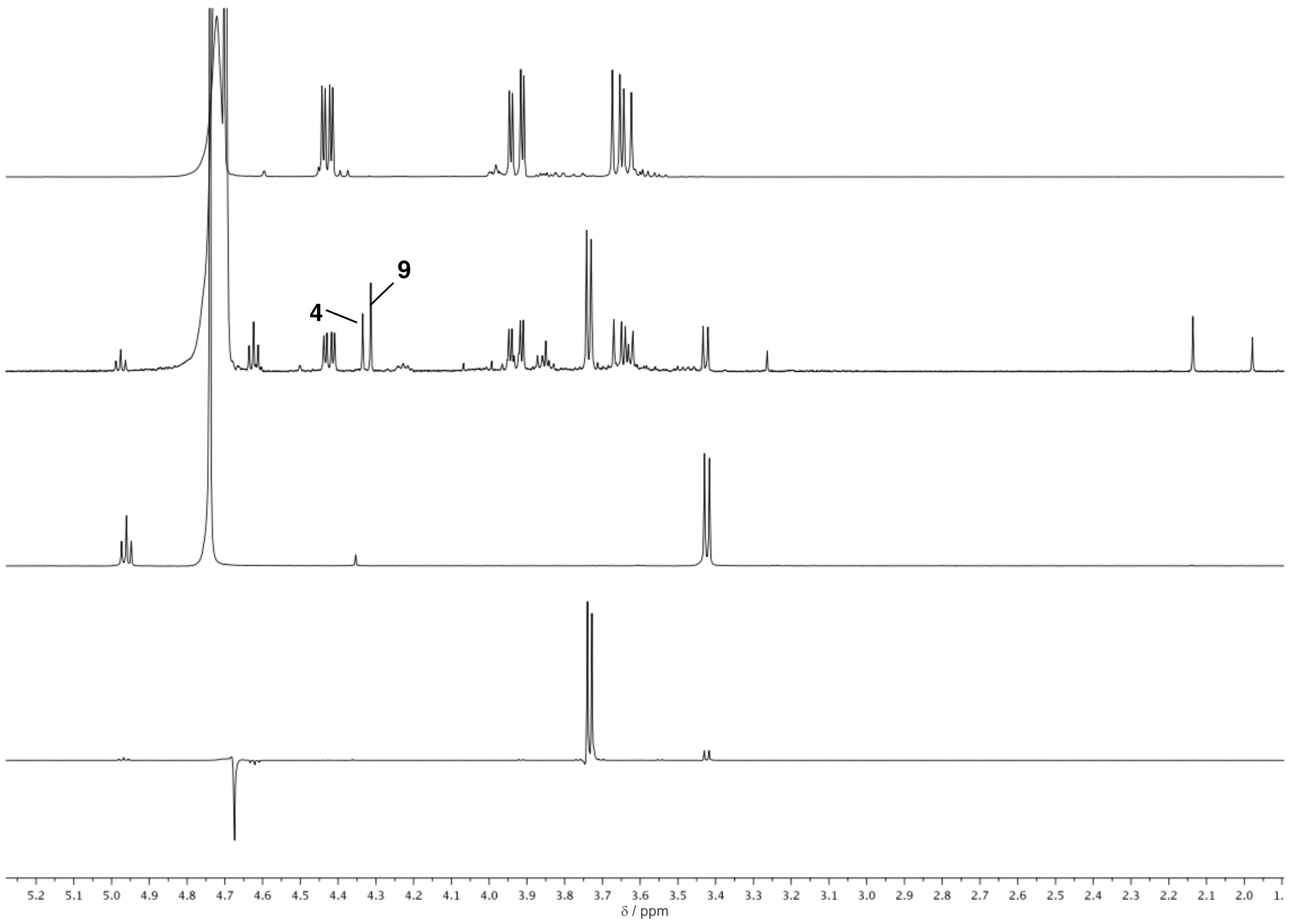

Supplementary Fig. 2 Photochemical reduction of glycolonitrile 4 and standards. Upper – ^{1}H NMR spectrum of glycolaldehyde-bisulfite adduct **7**; upper centre – ^{1}H NMR spectrum after 2 h irradiation of a solution of glycolonitrile (30 mM), Na_2SO_3 (60 mM), NaH_2PO_4 (100 mM) and $K_4[Fe(CN)]$ (5 mM) at pH 6.5; lower centre – ^{1}H NMR spectrum of commercial glycolaldehyde **5**; lower – ^{1}H NMR spectrum of glyceronitrile **6**.

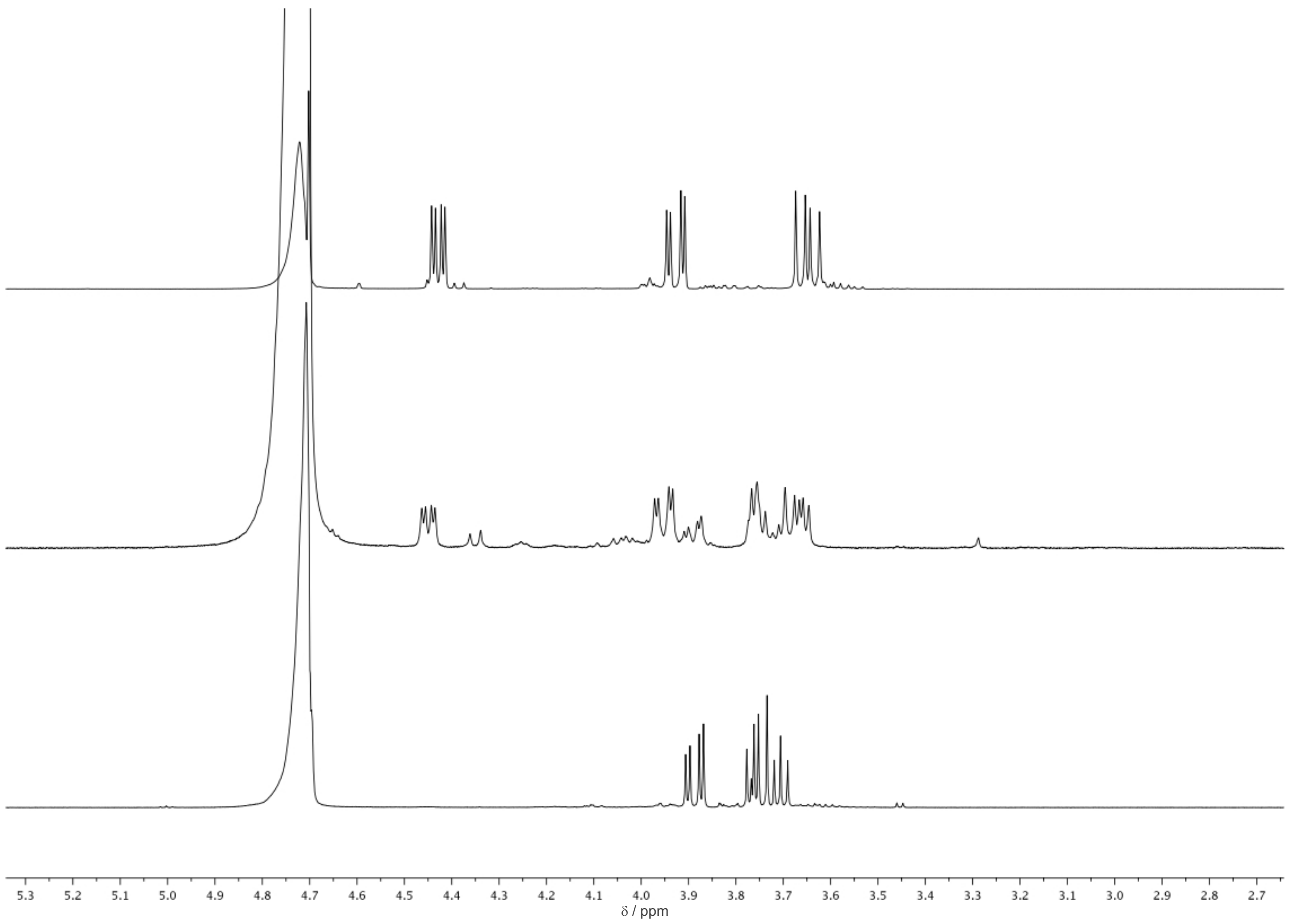

Supplementary Fig. 3 Reduction of 4 giving glycolaldehyde-bisulfite adduct 7 and standards. Upper – ^{1}H NMR spectrum of glycolaldehyde-bisulfite adduct **7**; centre – ^{1}H NMR spectrum after 1.5 h irradiation of a solution of glycolonitrile **4** (30 mM), Na_2SO_3 (150 mM), NaH_2PO_4 (100 mM) and $K_4[Fe(CN)]$ (5 mM) at pH of 6.5; Lower – ^{1}H NMR spectrum of the aminosulfonate adduct of glycolaldehyde **17** (glycolaldehyde **5** (50 mM), Na_2SO_3 (60 mM), c.NH_4OH (100 μL/mL) at pH 9.2). Slight variations in chemical shift are due to different pH of the solutions.

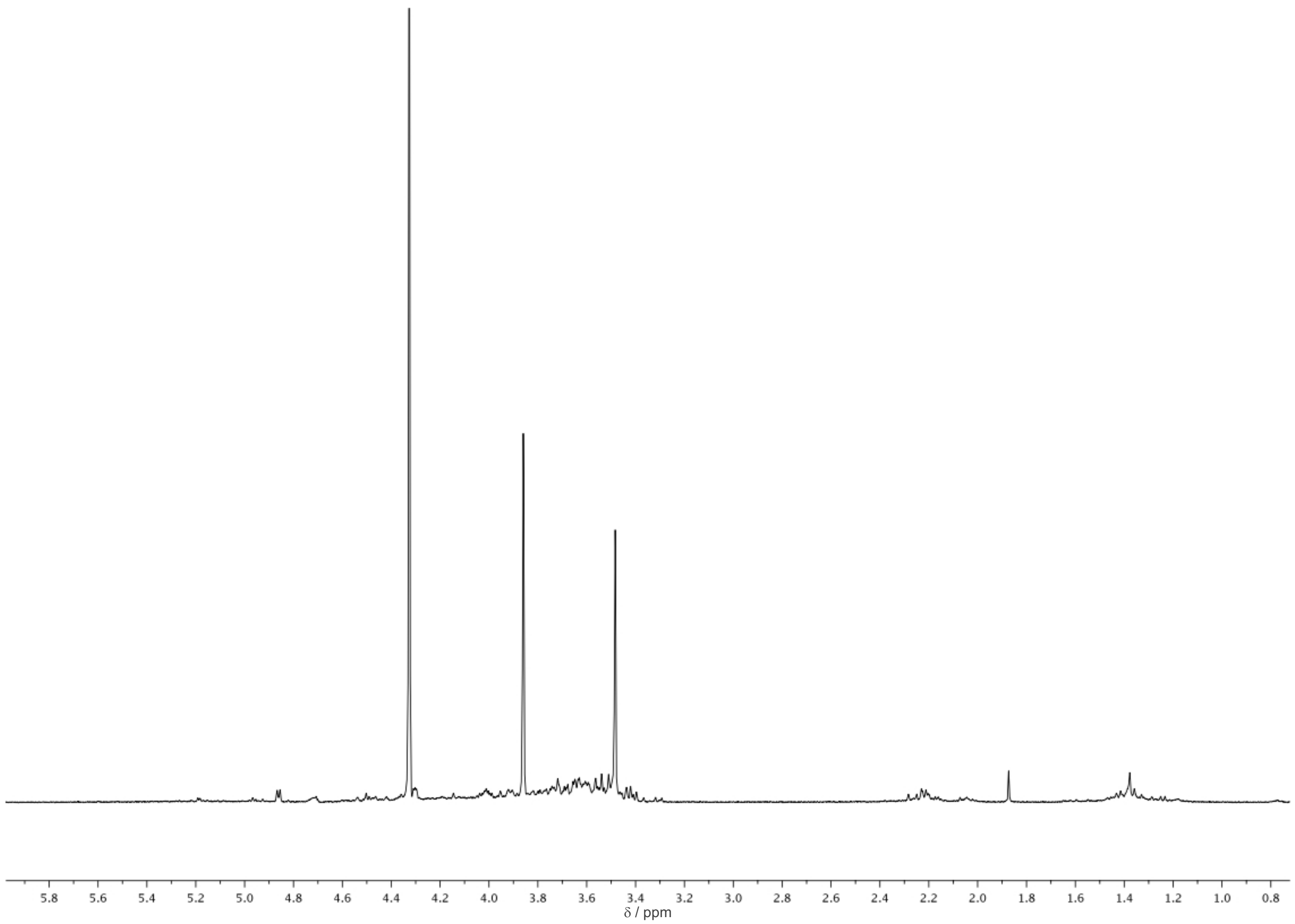

Supplementary Fig. 4 Enhancement of Fig. 4, panel c, lower spectrum (main text). ^{1}H NMR Spectrum solution of glyceraldehyde **10** and NaH_2PO_4 at pH 6.5 after heating to dryness for 40 h then redissolving in D_2O. Singlet observed at 4.35 ppm due to DHA **11**, singlet observed at 3.85 ppm due to glycolate, singlet observed at 3.48 due to the hydrate of **11**. Multiple small peaks result from oligomerisaiotn of **10**.

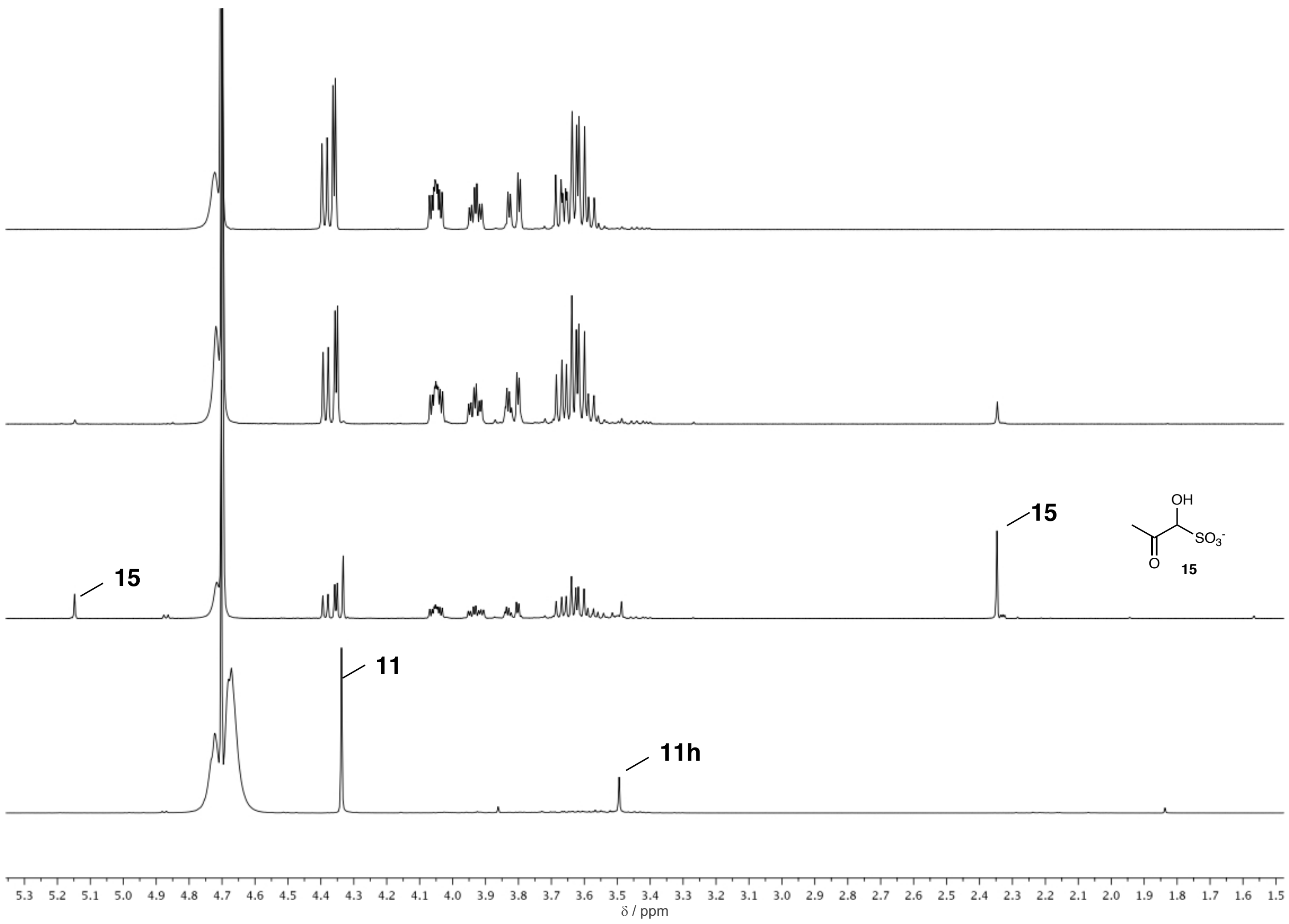

Supplementary Fig. 5 Protection of glyceraldehyde 10 by bisulfite. Upper – ^{1}H NMR spectrum of glyceraldehyde-bisulfite adduct **14** (glyceraldehyde **10** (100 mM), Na_2SO_3 (140 mM) and NaH_2PO_4 (100 mM) at pH 6.5); centre upper – as upper after heating at 55 ºC for 24 h; centre lower – as upper, after heating at 55 ºC for 2 weeks; lower – ^{1}H NMR spectrum of a solution of **10** (100 mM) and NaH_2PO_4 (100 mM) at pH 6.5 after heating at 55 ºC for 18 h. **11h** Denotes the hydrate of DHA **11**.

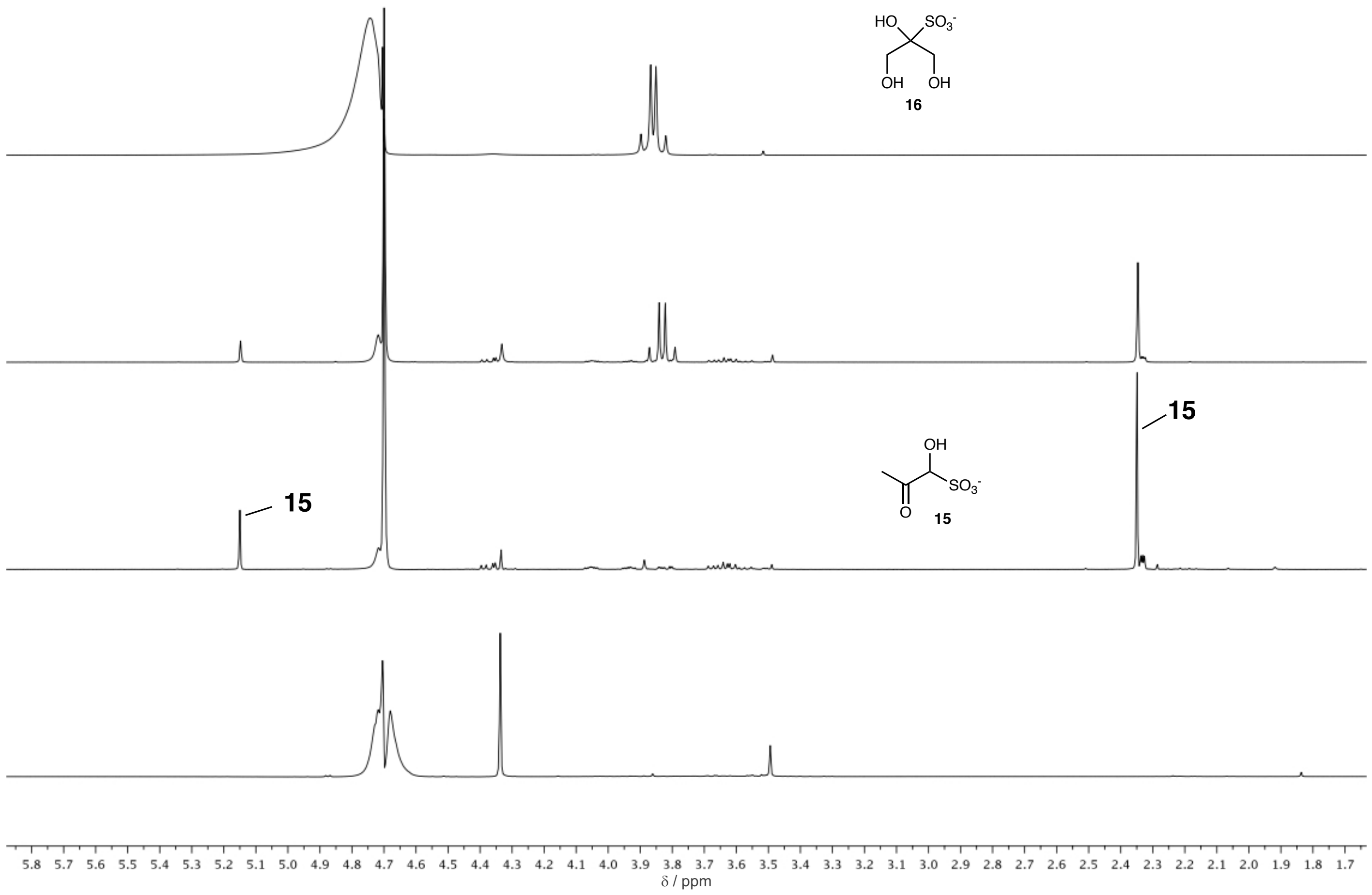

Supplementary Fig. 6 The fate of DHA-bisulfite adduct 11 when heated in solution. Upper – ^{1}H NMR spectrum of DHA-bisulfite adduct **16** (DHA (100 mM), Na_2SO_3 (140 mM) and NaH_2PO_4 (100 mM) at pH 6.5); centre upper – as upper after heating at 55 ºC for 24 h; centre lower – as upper, after heating at 55 ºC for 2 weeks; lower – ^{1}H NMR spectrum of a solution of DHA **11** (100 mM) and NaH_2PO_4 (100 mM) at pH 6.5 after heating at 55 ºC for 20 h.

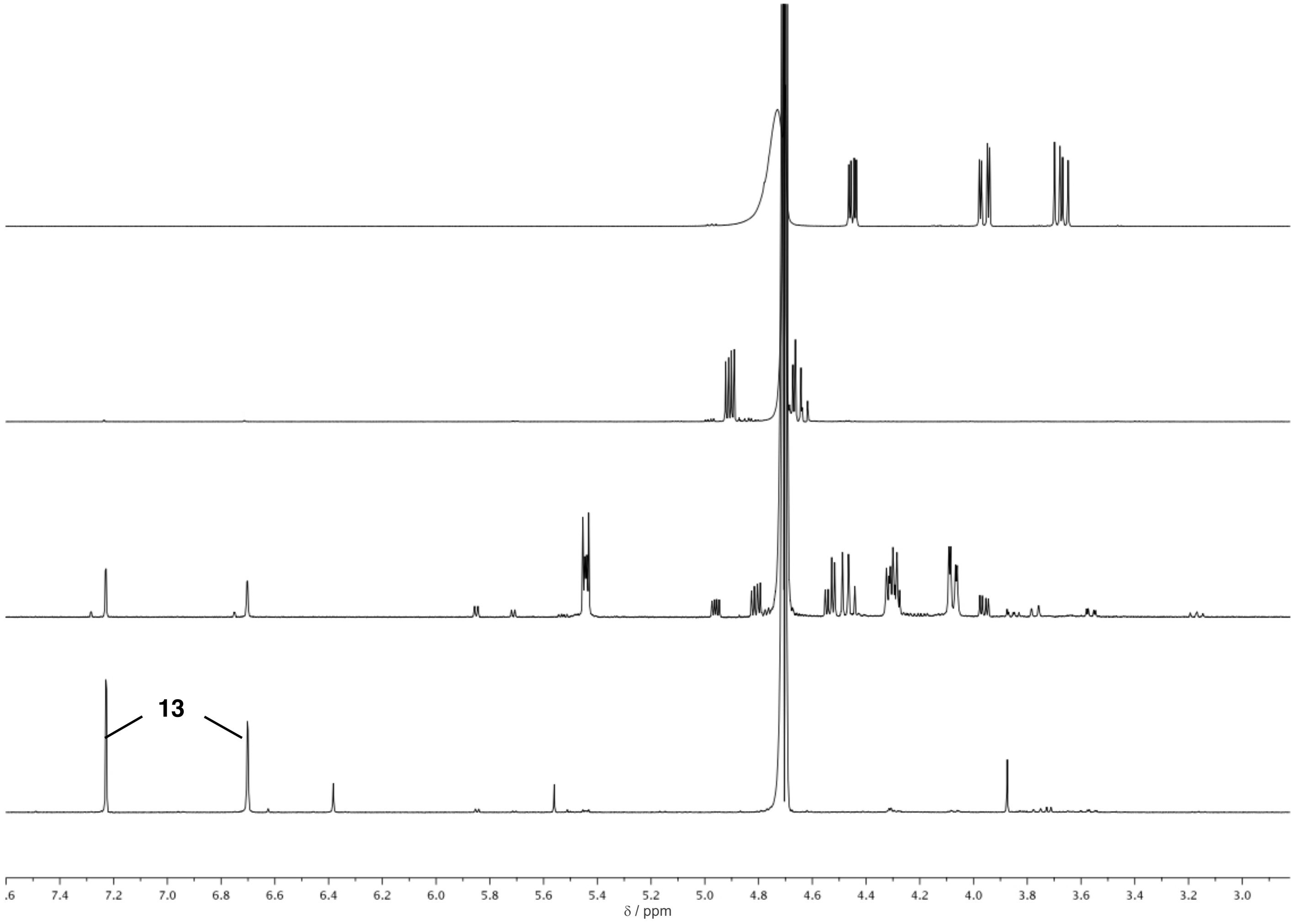

Supplementary Fig. 7 Glycolaldehyde-bisulfite 7 adduct reacting with NH_2CN *vs* CaNCN. Upper – ^1H NMR spectrum of glycolaldehyde **5** (50 mM), Na_2SO_3 (60 mM), cyanamide (175 mM) and NaH_2PO_4 (50 mM) heated at 45 ºC for ½ h; centre upper – as upper after heating at 45 ºC for 12 h; centre lower – ^1H NMR spectrum of glycolaldehyde **5** (50 mM), Na_2SO_3 (60 mM), CaNCN (175 mM) and NaH_2PO_4 (50 mM) heated at 45 ºC for ½ h; lower – as centre lower after heating at 45 ºC for 12 h.

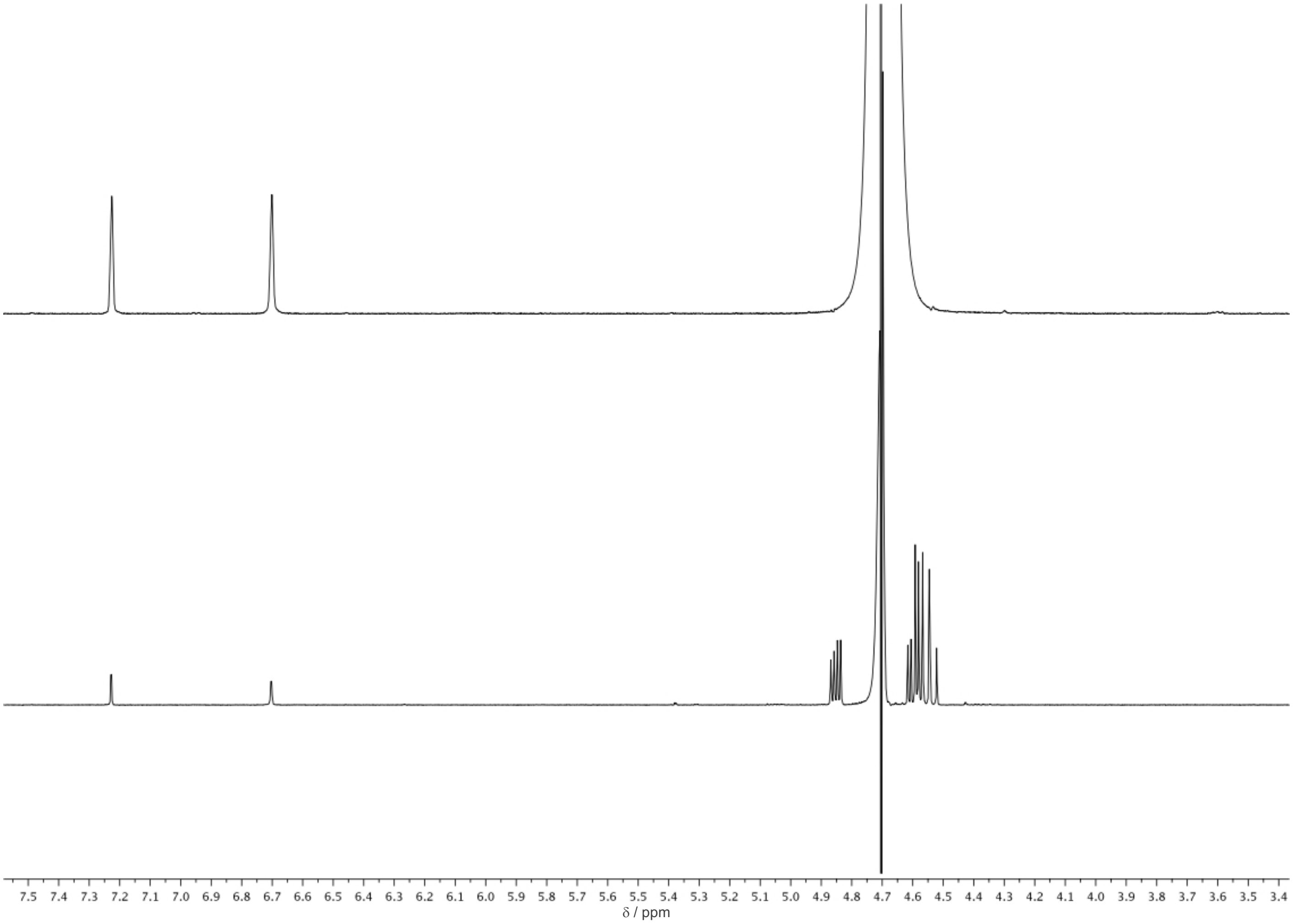

Supplementary Fig. 8 Reaction of 2-aminooxazole 13 with HSO_3^-. Upper – ^1H NMR spectrum of commercial 2-aminooxazole **13**; lower – ^1H NMR spectrum of a solution of **13** (50 mM), Na_2SO_3 (60 mM) and NaH_2PO_4 (50 mM) after heating at 45 °C for 18 h. As can be seen heating **13** with bisulfite leads to the same compound as heating glycolaldehyde-bisulfite adduct **7** with NH_2CN *cf.* Supplementary Fig. 7, centre upper (chemical shifts vary slightly due to small differences in the pH of the solutions).

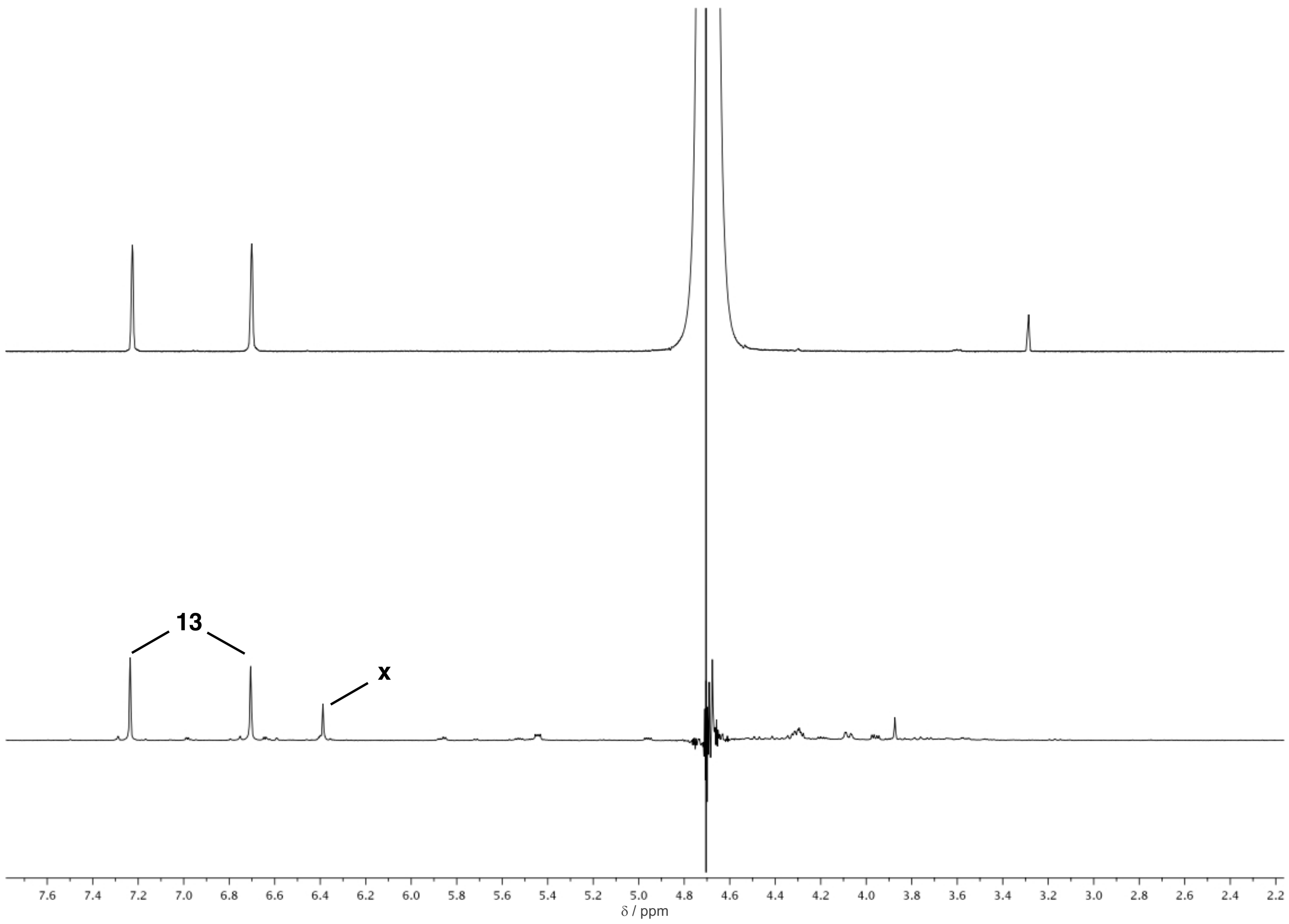

Supplementary Fig. 9 Uninterrupted synthesis of 2-aminooxazole 13 using flow equipment. Upper – ^{1}H NMR spectrum of commercial 2-aminooxazole **13**; lower – typical ^{1}H NMR spectrum of a solution of the crude reaction mixture at the end of the flow sequence where 2 streams are merged, one containing glycolonitrile **4** the other containing $NaHSO_3$, $K_4[Fe(CN)_6]$ and NaH_2PO_4. See Fig. 7, main text. Compound X was an unidentified by-product.

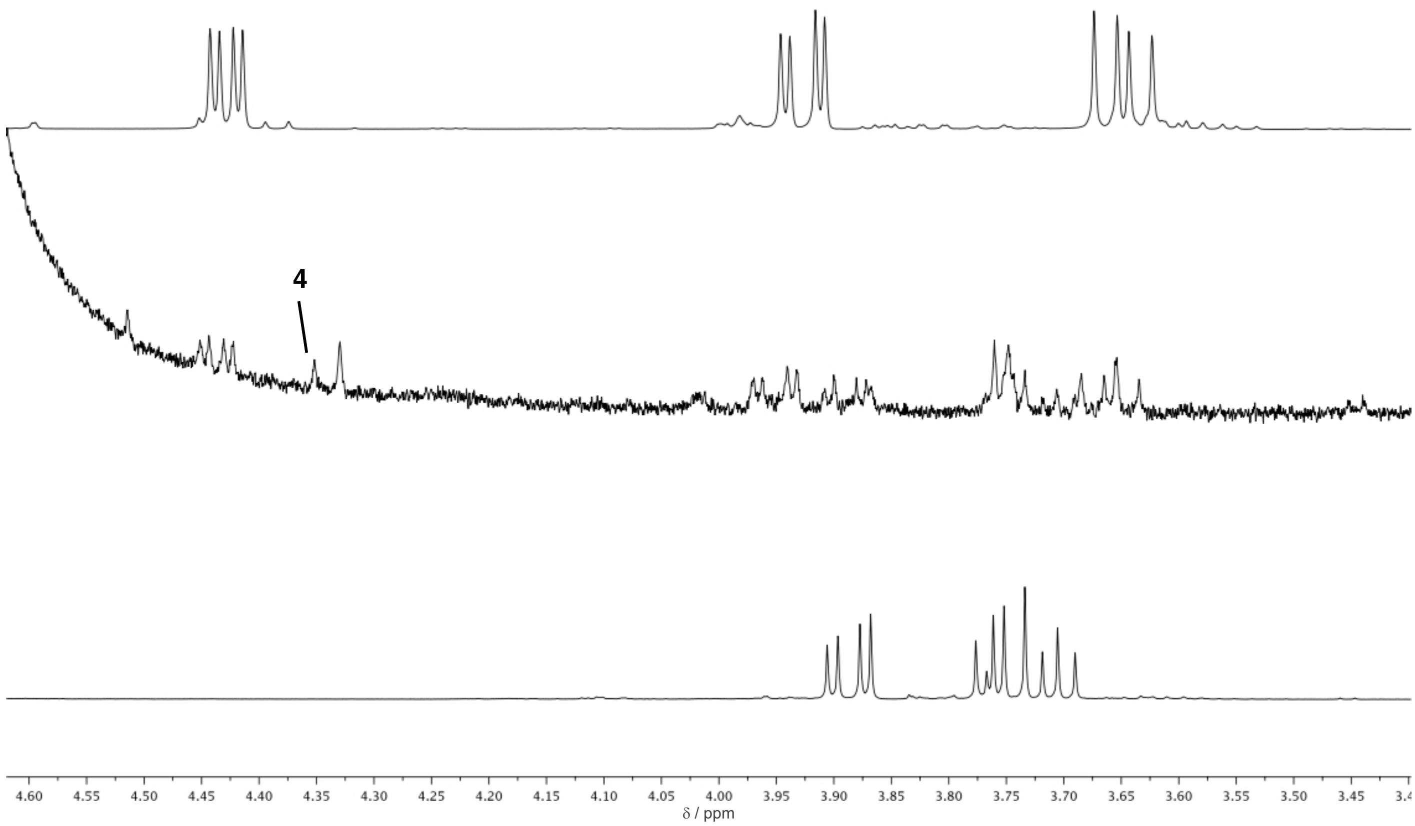

Supplementary Fig. 10 Reduction of glycolonitrile 4 at micromolar concentrations. Upper – ^{1}H NMR spectrum of glycolaldehyde-bisulfite adduct **7**; centre – ^{1}H NMR spectrum after 20 min irradiation of a solution of glycolonitrile **4** (0.5 mM), Na_2SO_3 (2.25 mM), NaH_2PO_4 (1 mM) and $K_4[Fe(CN)]$ (0.1 mM) at pH of 6.5. The large shoulder of a peak at the left-hand side is due to HOD; lower – ^{1}H NMR spectrum of the aminosulfonate adduct of glycolaldehyde **17** (glycolaldehyde **5** (50 mM), Na_2SO_3 (60 mM), c.NH_4OH (100 μL/mL) at pH 9.2). Slight variations in chemical shift are due to different pH of the solutions.

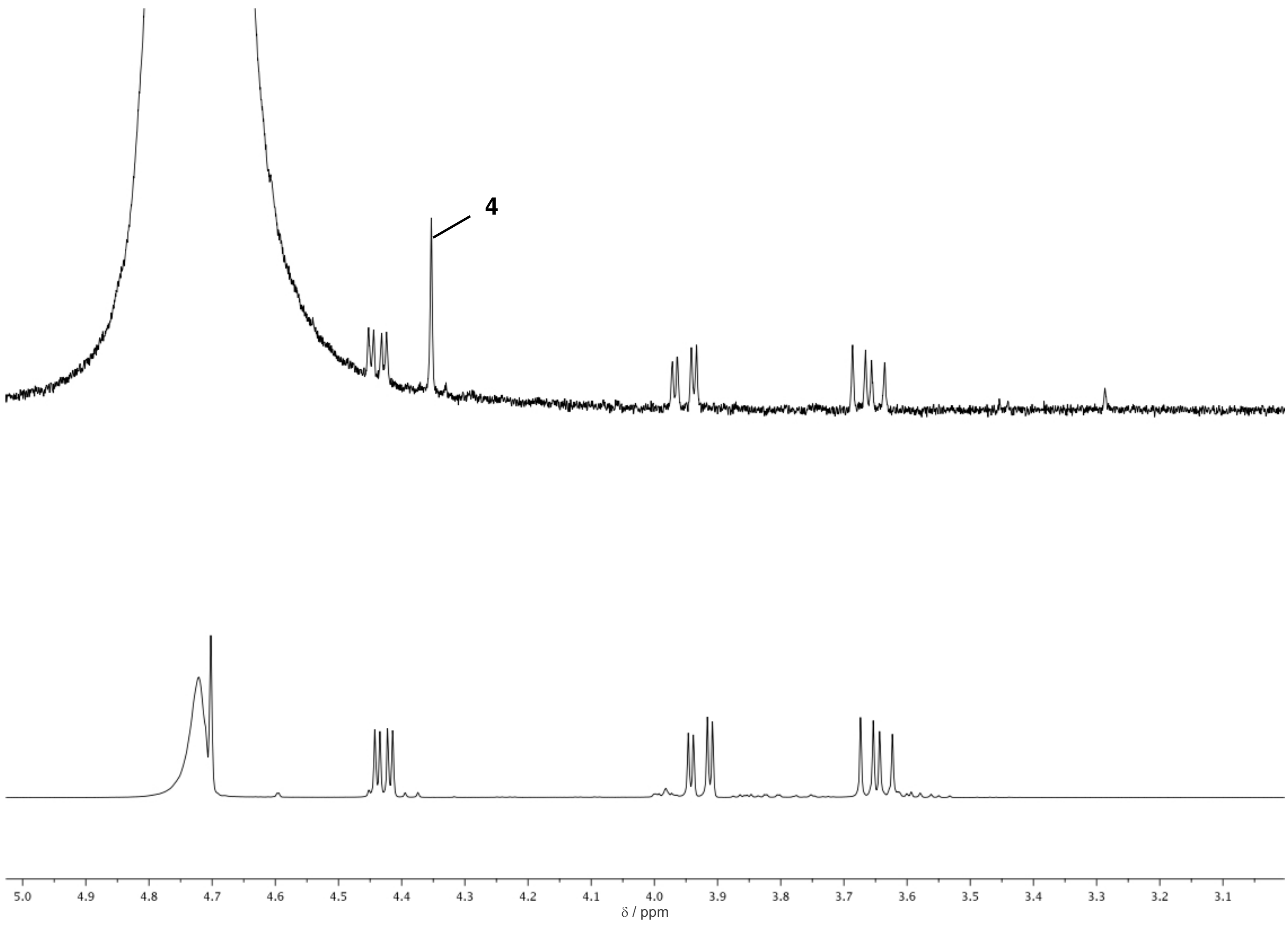

Supplementary Fig. 11 Reduction of glycolonitrile 4 at micromolar concentrations in absence of iron. Upper – ^{1}H NMR spectrum after 1 h irradiation of a solution of glycolonitrile **4** (0.5 mM) and Na_2SO_3 (2.25 mM) at pH of 6.5. The large peak at the left-hand side is due to HOD. Lower – ^{1}H NMR spectrum of glycolaldehyde-bisulfite adduct **7**.

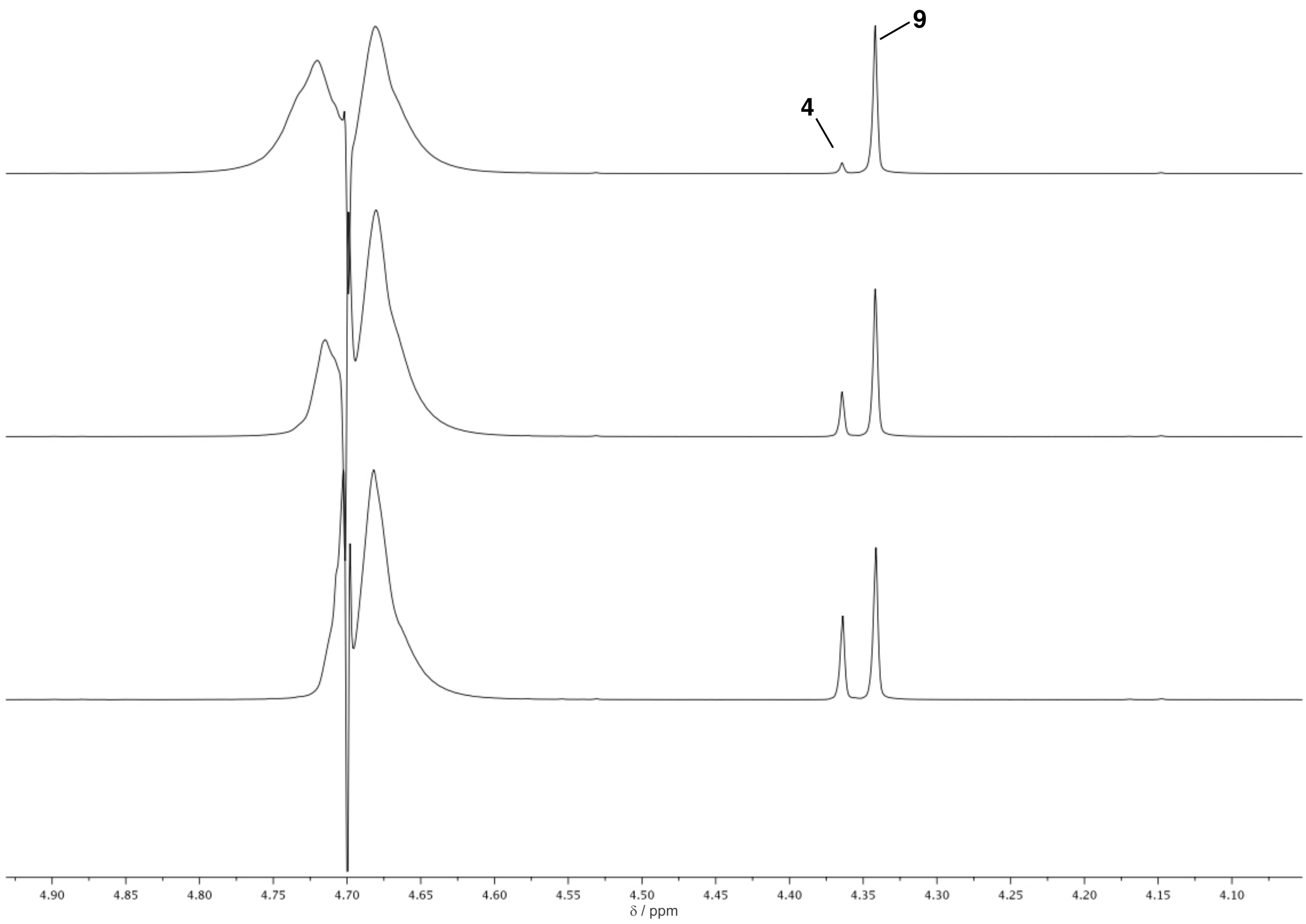

Supplementary Fig. 12 Exchange of HCN and HSO_3^- starting from HMSA 9 at neutral pH. Upper – ^{1}H NMR spectrum after 15 min; centre – ^{1}H NMR spectrum after 2 d; lower – ^{1}H NMR spectrum after 7 d.

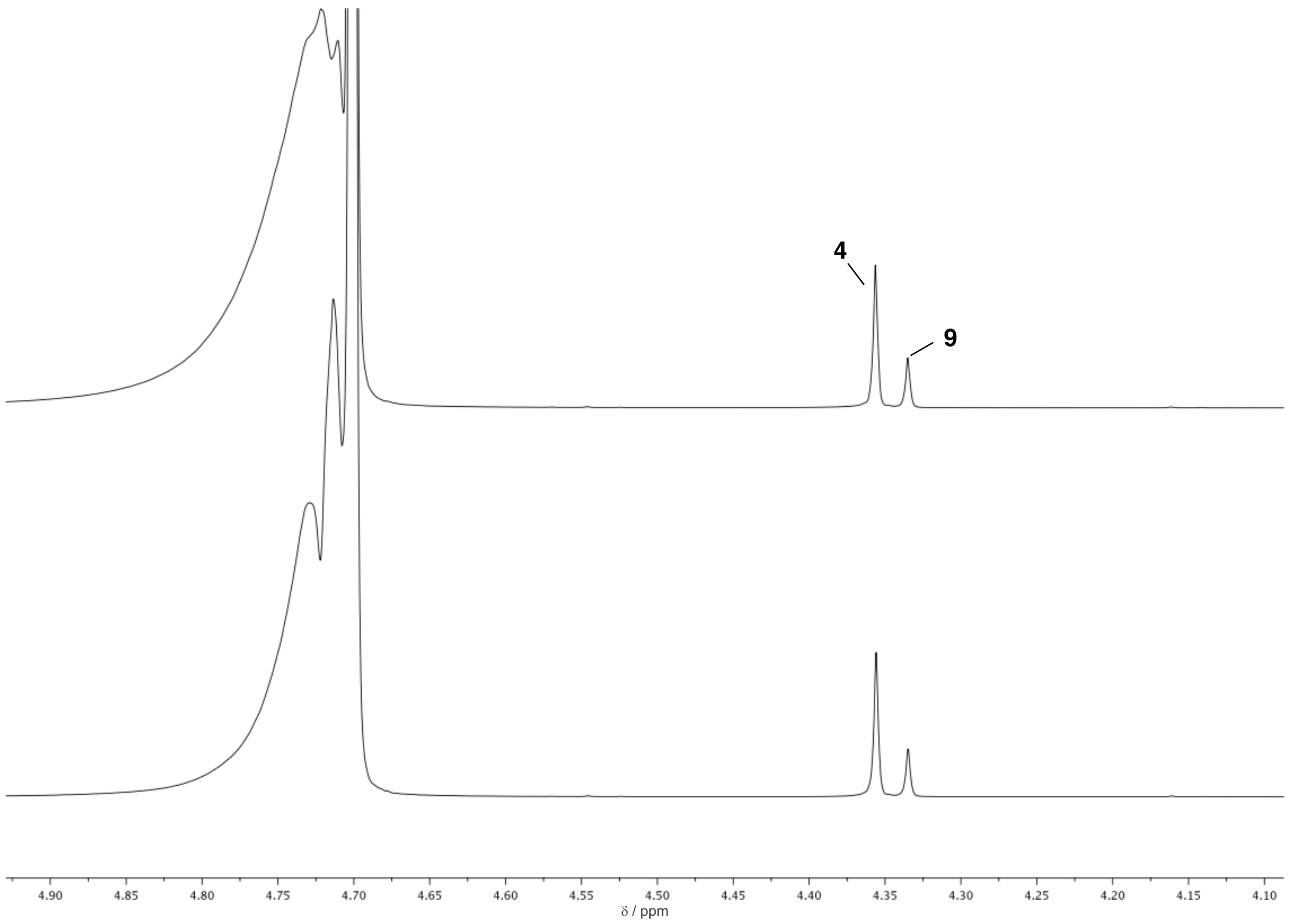

Supplementary Fig. 13 Exchange of ^{-}CN and SO_3^{2-} starting from HMSA 9 at pH 9.5. Upper – ^{1}H NMR spectrum after 15 min; lower – ^{1}H NMR spectrum after 24 h.

Supplementary references

1. Decker, P., Schweer, H. & Pohlmann, R. Bioids: X. Identification of formose sugars, presumably prebiotic metabolites, using capillary gas chromatography/gas chromatography-mass spectrometry of *n*-butoxime trifluoroacetates on OV-225. *J. Chromatography A* **244**, 281-291 (1982).
2. Vázquez-Mayagoitia, Á. *et al.* On the stabilization of ribose by silicate minerals. *Astrobiol.* **11**, 115-121 (2011).
3. Kim, H-J. *et al.* Synthesis of carbohydrates in mineral-guided prebiotic cycles. *J. Am. Chem. Soc.* **133**, 9457-9468 (2011).
4. Todd, Z. R. *et al.* Solvated-electron production using cyanocuprates is compatible with the UV-environment on a Hadean-Archaean Earth. *Chem. Commun.* **54**, 1121-1124 (2018).
5. Kim, H-J. *et al.* Evaporite borate-containing mineral ensembles make phosphate available and regiospecifically phosphorylate ribonucleosides: borate as a multifaceted problem solver in prebiotic chemistry. *Angew. Chem. Int. Ed.* **55**, 15816-15820 (2016).
6. Reid, C. & Orgel, L. E. Synthesis of sugars in potentially prebiotic conditions. *Nature*, **216**, 455 (1967).
7. Gabel, N. W. & Ponnamperuma, C. Model for origin of monosaccharides. *Nature,* **216**, 453-455 (1967).
8. Shapiro, R. Prebiotic ribose synthesis: a critical analysis. *Orig. Life Evol. Biosph.* **18**, 71-85 (1988).
9. Cleaves II, H. J. Formose reaction. *Encyclopedia of Astrobiology* (Springer, Berlin, Heidelberg, 2011).
10. Löb, W. Uber das verhalten des formamids unter der wirkung der stillen entladung ein beitrag zur frage der stickstoff-assimilation. *Ber. Deutch. Chem. Ges.* **46**, 684-697 (1913).
11. Shapiro, R. A simpler origin for life. *Sci. Am.* (February 12, 2007).
12. Benner, S. A., Kim, H-J. & Carrigan, M. A. Asphalt, water and the prebiotic synthesis of ribose, ribonucleosides, and RNA. *Acc. Chem. Res.* **45**, 2025-2034 (2012).

13. Hazen, R. M. Paleomineralogy of the Hadean eon: a preliminary species list. *Am. J. Sci.* **313**, 807-843 (2013).

14. Angyal, S. J. The Lobry de Bruyn-Alberda van Ekenstein transformation and related reactions. *Top. Curr. Chem. Glycoscience*, **215**, 1 (2001).

The following references are cited in Supplementary ref. 14 and pertain to borate and the isomerisation of sugars:

15. Mendicino, J. F. Effect of borate on the alkali-catalysed isomerisation of sugars. *J. Am. Chem. Soc.* **82**, 4975-4979 (1960).

16. Carubelli, R. Transformation of disaccharides during borate ion-exchange chromatography. Isomerization of lactose into lactulose. *Carbohydr. Res.* **2**, 480-485 (1966).

17. Hicks, K. B. & Parrish, F. W. A new method for the preparation of lactulose from lactose. *Carbohydr. Res.* **82**, 393-397 (1980).

18. De Bruijn, J. M., Kieboom, A. P. G. & van Bekkum, H. Alkaline degradation of monosaccharides III. Influence of reaction parameters upon the final product composition. *Recl. Trav. Chim. Pays-Bas* **105**, 176-183 (1986).
